# Background-Free, Label-Free, and Reproducible SERS via Plasma–Halide-Engineered Gold Nanoparticles

**DOI:** 10.1021/acsmeasuresciau.5c00196

**Published:** 2026-05-04

**Authors:** Yen Ling Chiu, Ya Chi Chen, Loid Elizabeth Urtecho Navas, Wei-Hung Chiang

**Affiliations:** 1 Department of Chemical Engineering, 34878 National Taiwan University of Science and Technology, Taipei 10607, Taiwan; 2 Sustainable Electrochemical Energy Development (SEED) Center, 34878 National Taiwan University of Science and Technology, Taipei City 10607, Taiwan

**Keywords:** surface-enhanced Raman scattering, nanoparticles, plasma synthesis, nanotechnology, sensor

## Abstract

Surface-enhanced Raman spectroscopy (SERS) is often limited by substrate-derived background from organic ligands (e.g., citrate), which degrades spectral fidelity, sensitivity, and quantitation. We report a measurement-oriented SERS platform based on size-tuned, microplasma-synthesized gold nanoparticles (AuNPs, 14–100 nm) that are surface-purified with bromide (Br^–^). Bromide competitively displaces weakly bound citrate while remaining Raman-inactive, thereby suppressing baseline features in the 1000–1800 cm^–1^ fingerprint region without introducing new peaks. An optimal Br^–^ window (∼1.5 mM, ∼3 h) achieves effective cleaning while avoiding surface passivation. The resulting substrates deliver strong enhancement (EF >10^6^), subnanomolar limits of detection (10^–9^ M for rhodamine 6G; 10^–10^ M for malachite green), and wide linear ranges (10^–9^–10^–4^ and 10^–10^–10^–4^ M, respectively). Spot-to-spot variability is low (RSD <∼8%), and activity is retained over weeks (>90% signal after storage), underscoring practical reproducibility and stability. Spectroscopic and surface charge analyses corroborate ligand displacement and improved adsorption on clean Au surfaces while preserving plasmonic response. This simple, green, and scalable plasma–halide strategy provides background-free, label-free, and reproducible SERS measurements. Driven by electrostatic complementarity, this platform offers a highly effective route for the quantitative trace analysis of cationic analytes, relevant to environmental monitoring and chemical safety.

## Graphical abstract

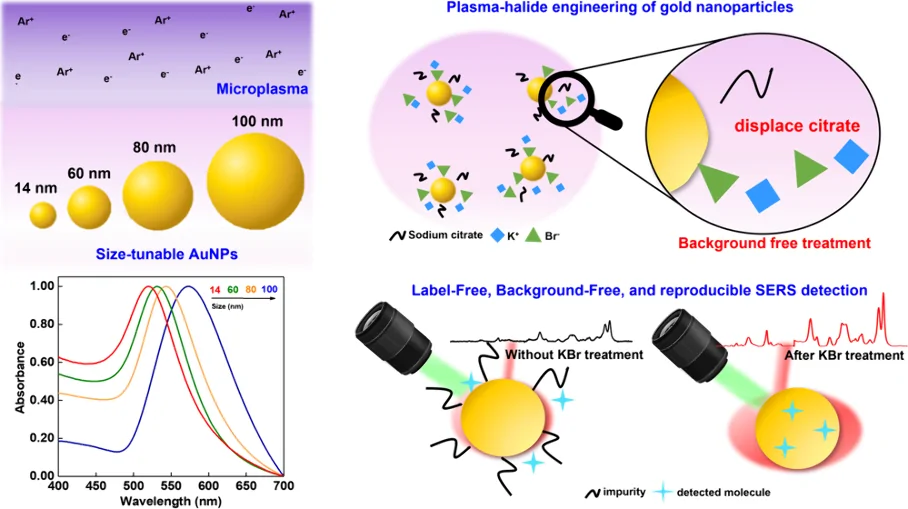

## 1 Introduction

Surface-enhanced Raman spectroscopy (SERS) is a powerful analytical technique that enables ultrasensitive label-free molecular detection with fingerprint-level specificity. Its applications span numerous disciplines, including biosensing, catalysis, optoelectronics, cancer diagnostics, and environmental monitoring.^1–4^ Recently, the design of advanced plasmonic arrays and hybrid nanostructures has further improved SERS sensitivity and specificity.^5,6^ These engineered substrates, often combined with molecular enrichment strategies, have proven highly effective for the trace detection of complex targets like biomolecules, multiple drugs, and environmental pollutants.^7,8^ However, the widespread implementation of SERS is hindered by one persistent challenge: background Raman signals originating from impurities or stabilizing agents on the surfaces of SERS-active nanomaterials. These signals often overlap with analyte peaks, reduce enhancement factors, and hinder reproducibility, particularly in the label-free detection of weakly scattering biomolecules, such as target proteins and cancer-associated exosomes.^5,9–13^ Consequently, the development of background-free SERS-active materials with high enhancement efficiency is critical. Such materials offer clean spectral baselines and enhanced analyte–surface interactions, thereby enabling precise and reproducible detection. Advancing this area is essential for unlocking the next generation of SERS applications across disciplines, from probing molecular dynamics to enabling clinically relevant diagnostics.

Conventional strategies to mitigate this issue often involve postsynthetic ligand exchange or multistep composite nanostructure assembly, which complicates synthesis, reduces scalability, and introduce batch-to-batch variability.^14^ For example, wet-chemical synthesis of SERS-active materials such as silver (Ag) and gold (Au) nanoparticles (NPs) and residual capping agents such as citrate often remain adsorbed on the nanoparticle surface, leading to strong background Raman signals and poor analyte adsorption, both of which significantly hinder the performance of SERS, especially for label-free detection.^15–17^ Furthermore, the synthesis process often requires lengthy reaction times, elevated temperatures, and toxic reagents, making it unsuitable for the scalable or green fabrication of high-quality SERS substrates. Although various strategies, including multistep washing, ligand exchange, and hybrid material integration, have been developed to address this issue, they typically involve complex procedures, harsh reagents, or lack scalability.^18^ Moreover, a few approaches simultaneously resolve the key challenges of surface cleanliness, plasmonic optimization, and environmentally sustainable synthesis.

Low-temperature plasma-assisted synthesis, particularly with atmospheric-pressure microplasmas, has emerged as a green, rapid, and versatile alternative to conventional wet-chemical methods. Microplasma systems generate a rich mixture of reactive species, including electrons, ions, and radicals, which can drive the reduction and nucleation processes without the need for harsh reductants or stabilizers. This enables the synthesis of metallic nanoparticles under mild, solvent-compatible, and environmentally benign conditions, greatly reducing waste and energy consumption. In addition, plasma-generated reactive species can modulate reduction kinetics and nucleation density during nanoparticle synthesis. Unlike traditional methods that often require elevated temperatures, toxic reagents, or lengthy reaction times, plasma synthesis offers precise control over particle size, morphology, and surface chemistry within minutes.^19^ These advantages are particularly beneficial for SERS-active materials, where surface purity, size uniformity, and plasmonic properties must be finely tuned to ensure strong and reproducible Raman enhancement.^20–24^

Here, we report a synergistic plasma–halide surface engineering strategy for the first-time fabrication of background-free, SERS-active AuNPs with tunable sizes and clean surfaces (Scheme 1). This approach uniquely combines microplasma-assisted green synthesis and halide-based surface purification, eliminating the need for harsh chemicals or multimaterial constructs while preserving high SERS activity. Using an ambient microplasma-assisted synthesis platform, we achieved rapid, green fabrication of AuNPs with controlled diameters (14–100 nm), followed by solution-based bromide ion (Br^–^) treatment to selectively displace Raman-active surface contaminants. Importantly, Br^–^ itself is Raman-inactive, enabling spectral background suppression without introducing new signals, which is a critical and often overlooked requirement in SERS applications. The resulting substrates exhibited strong SERS activity, enabling subnanomolar detection of environmental analytes, such as rhodamine 6G (R6G) and malachite green (MG), while maintaining high signal reproducibility and long-term stability. This work represents the first demonstration of using a microplasma–halide dual approach to produce SERS-active AuNPs that are simultaneously surface-purified, plasmon-tuned, and scalable without relying on additional material coatings or multistep assembly. Our findings not only establish a simple yet powerful paradigm for the preparation of low-background SERS substrates, uniting green synthesis, structural tunability, and analytical sensitivity but also offer broad potential for biomedical sensing, environmental monitoring, and point-of-care (POC) diagnostics.

**Scheme 1:**
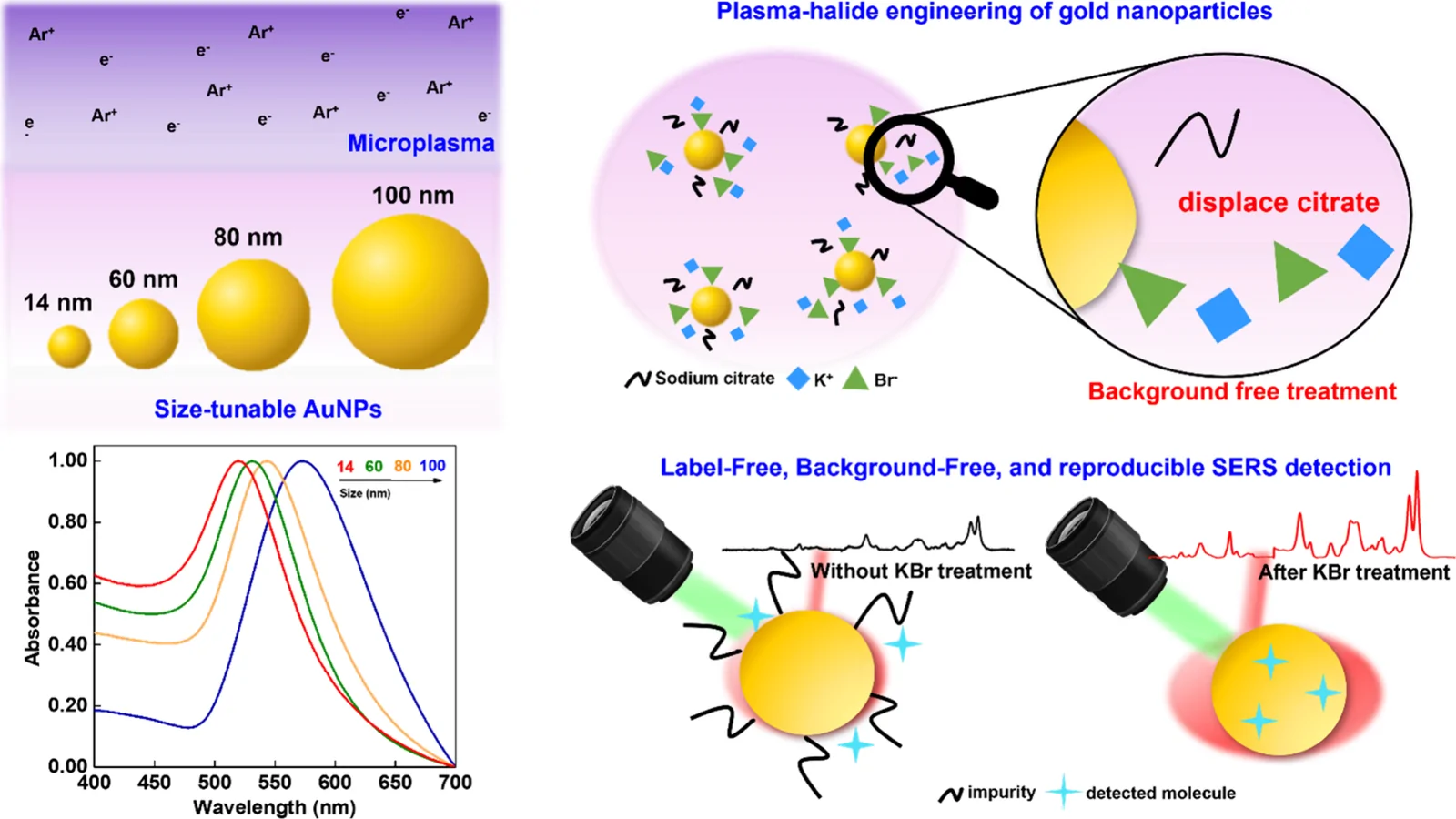
Two-Step Synthesis of Clean-Surface, Size-Tunable Gold Nanoparticles (AuNPs) for Low-Background SERS Detection of Environmental Pollutants^a^

a AuNPs of tunable sizes were rapidly synthesized via an ambient microplasma technique, followed by potassium bromide (KBr) treatment to eliminate surface-bound citrate and suppress background signals. The halide-treated AuNPs exhibited enhanced SERS performance due to improved electrostatic interactions with target analytes.

## 2 Results and Discussion

### 2.1 Synthesis and Characterization of Plasma-Engineered AuNPs

Figure 1 presents the morphological characterization of AuNPs synthesized via the microplasma-assisted approach. Particle size tuning was achieved by modulating two key parameters: the sodium citrate (SC) concentration and the argon (Ar) gas flow rate. AuNPs with average diameters of 60, 80, and 100 nm were obtained by decreasing the citrate concentration to 10, 5, and 2 mM, respectively, while maintaining a constant argon flow rate of 25 standard cubic centimeters per minute (sccm). In contrast, 14 nm AuNPs were synthesized by increasing the argon flow rate to 40 sccm while maintaining a citrate concentration of 10 mM. Scanning electron microscopy (SEM) and transmission electron microscopy (TEM) confirmed that the synthesized AuNPs were predominantly spherical with narrow size distributions. TEM images of the 14 nm AuNPs at low (80k×) and high (200k×) magnifications (Figure 1a,b) showed well-dispersed particles without noticeable aggregation. High-resolution TEM (HRTEM) analysis (Figure 1c) revealed clear lattice fringes with an interplanar spacing of 0.23 nm, corresponding to the (111) planes of face-centered cubic (fcc) gold, consistent with previous reports.^25^ Size distribution analysis based on TEM (Figure 1d) indicated an average particle diameter of 14.3 ± 0.4 nm. For the larger AuNPs, SEM-based measurements confirmed average diameters of 59.3 ± 7.5, 79.2 ± 8.5, and 106.9 ± 13.4 nm (Figure 1e–h), validating successful size control through citrate concentration adjustment under plasma treatment.

**Figure 1:**
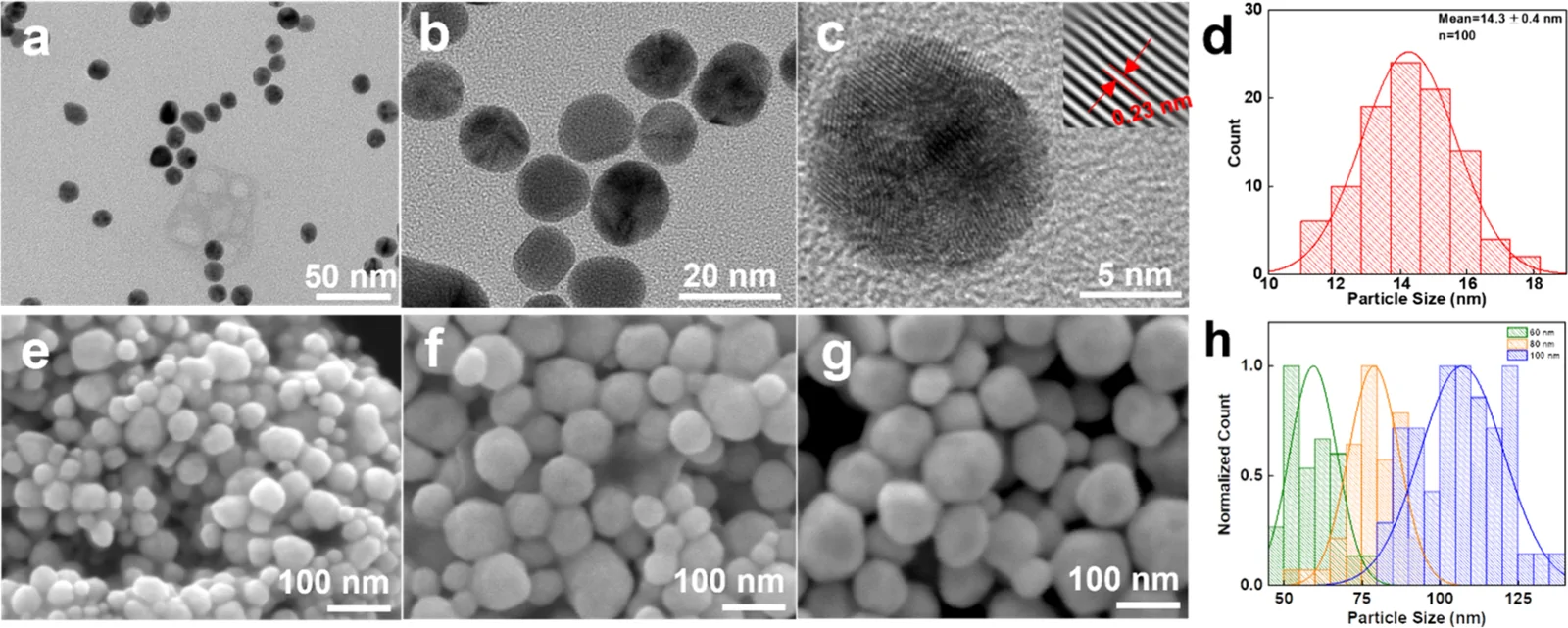
Plasma-size-controlled synthesis and morphological characterization of AuNPs. (a, b) TEM images of 14 nm AuNPs at low (80k×) and high (200k×) magnification, showing uniform dispersion without aggregation. (c) High-resolution TEM (HRTEM) image of a single AuNP, revealing a lattice fringe spacing of 0.23 nm corresponding to the Au(111) plane. (d) Size distribution histogram of 14 nm AuNPs derived from TEM analysis. (e–g) SEM images of AuNPs synthesized at varying sodium citrate concentrations, yielding average diameters of 60, 80, and 100 nm, respectively. (h) Corresponding particle size distributions from SEM analysis confirm successful size tuning through modulation of citrate concentration and argon flow rate.

To investigate the relationship between the nanoparticle size and optical response, the plasmonic properties of the synthesized AuNPs were examined using UV–vis absorbance spectroscopy (Figure 2). The spectra exhibited well-defined and symmetric surface plasmon resonance (SPR) bands that progressively red-shifted with increasing particle diameter. Specifically, the absorption maxima were located at 520, 531, 543, and 573 nm for AuNPs with average sizes of 14, 60, 80, and 100 nm (from the TEM and SEM data) (Figure 2a), respectively. These red shifts are consistent with the expected size-dependent SPR behavior of gold nanostructures and align well with the size measurements obtained from TEM and SEM analyses. The close correlation between particle size and optical response underscores the effectiveness of the microplasma-assisted synthesis in controlling both the morphology and plasmonic characteristics of AuNPs, making them useful for SERS applications.

**Figure 2:**
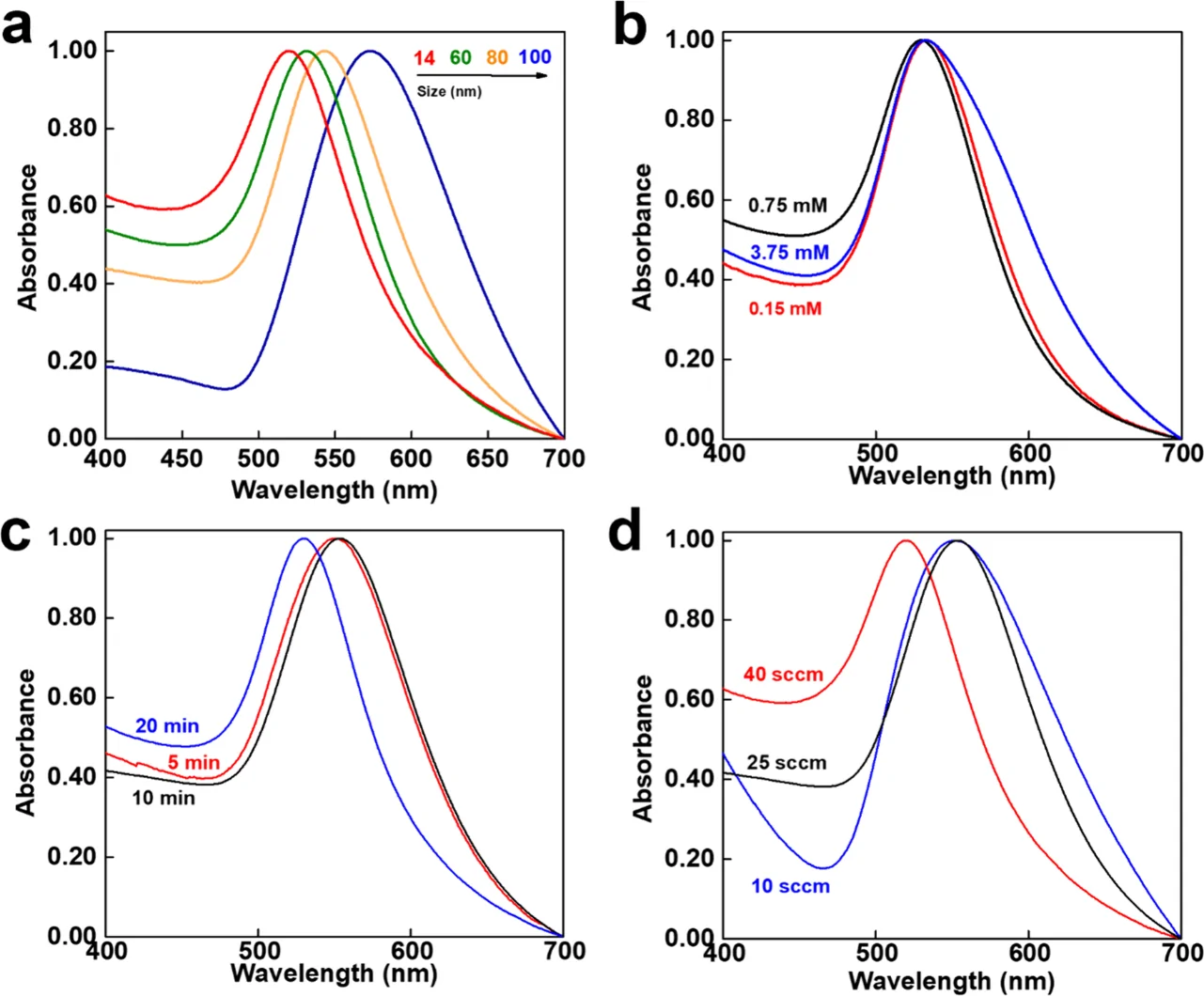
UV–vis absorbance spectra of AuNPs synthesized via microplasma under varying conditions. (a) Particle size-dependent plasmon resonance of AuNPs with diameters of 14, 60, 80, and 100 nm, showing red-shifted SPR peaks with increasing size. (b) Effect of varying HAuCl_4_ precursor concentrations (0.15 and 0.75 mM) on SPR peak intensity and nanoparticle formation. (c) Influence of microplasma treatment duration on AuNP optical response, highlighting improved growth and crystallinity with extended reaction time. (d) Effect of Ar gas flow rate on plasmonic behavior, where higher flow rates lead to smaller particle sizes and blue-shifted SPR peaks.

The above results can be rationalized based on classical nucleation and growth principles as well as the specific characteristics of plasma–liquid interactions. At higher citrate concentrations, more capping ligands are available to stabilize the nascent nuclei, favoring the formation of a large number of smaller particles by suppressing growth (Figure 2a). Conversely, lower citrate concentrations provide less surface passivation, allowing fewer nuclei to grow into larger particles owing to reduced steric and electrostatic stabilization. Additionally, increasing the argon flow rate enhanced the generation of reactive plasma species (e.g., hydrated electrons and radicals), accelerating the reduction kinetics of the Au^3+^ ions (Figure 2d). It is important to note that the effect of gas flow on reactive species generation is highly dynamic and closely dependent on the experimental configuration. While in some plasma systems an increased flow might dilute reactive species, in our atmospheric-pressure microplasma setup, higher argon flow rates actively modify the hydrodynamic interactions between the plasma plume and the liquid surface. This change alters the electron flux density and enhances convective mass transfer at the gas–liquid interface, which consequently promotes the continuous generation of the observed reactive species (e.g., hydrated electrons and radicals).^19,26,27^ This leads to higher supersaturation, promoting burst nucleation and ultimately yielding smaller AuNPs. Thus, particle size control in this system arises from the delicate balance between plasma-induced reduction rates and citrate-mediated growth stabilization. To further verify the critical role of the microplasma step, a control experiment was conducted using the conventional citrate reduction method with identical precursor concentrations (0.75 mM HAuCl_4_ and 2 mM sodium citrate). As shown in Figure S2a, the absorption spectrum recorded after 20 min of reaction lacks a distinct surface plasmon resonance peak. This indicates that nanoparticle formation is still incomplete at this stage. A characteristic plasmon peak only appears at approximately 545 nm after 2 h of reaction (Figure S2b), confirming the eventual formation of citrate-reduced gold nanoparticles. In contrast, the microplasma process achieves rapid nanoparticle formation within just 20 min. This comparison directly highlights the essential role of the microplasma in accelerating nucleation and growth. The microplasma method enables the fine-tuning of these parameters under ambient conditions, offering precise and reproducible control over nanoparticle morphology without requiring harsh reagents or elevated temperatures. This level of control is crucial for optimizing SPR properties and, consequently, SERS enhancement performance.

### 2.2 Micro-Raman Spectroscopy Study of Plasma–Halide-Engineered AuNPs

To study the intrinsic background signals of AuNPs synthesized via microplasma, Raman spectroscopy was conducted over the spectral range of 500–3000 cm^–1^. As shown in Figure 3a (with a representative spectrum of 14 nm AuNPs in Figure S4), all AuNP samples exhibited noticeable background features within the 1000–1800 cm^–1^ region. This spectral range, commonly referred to as the molecular fingerprint region, contains key vibrational modes such as aromatic ring breathing, C=O stretching, and C–N/C–C skeletal vibrations, which are critical for the accurate identification of molecular species in SERS-based biosensing, drug monitoring, and label-free diagnostics.^18,28,29^ However, background signals originating from residual surface ligands, such as citrate or partially reduced by products from plasma–liquid reactions, can overlap with these vibrational modes and compromise the detection of weak analyte signals. Such interference diminishes both the spectral clarity and analytical sensitivity, which is particularly detrimental for trace-level or label-free detection.

**Figure 3:**
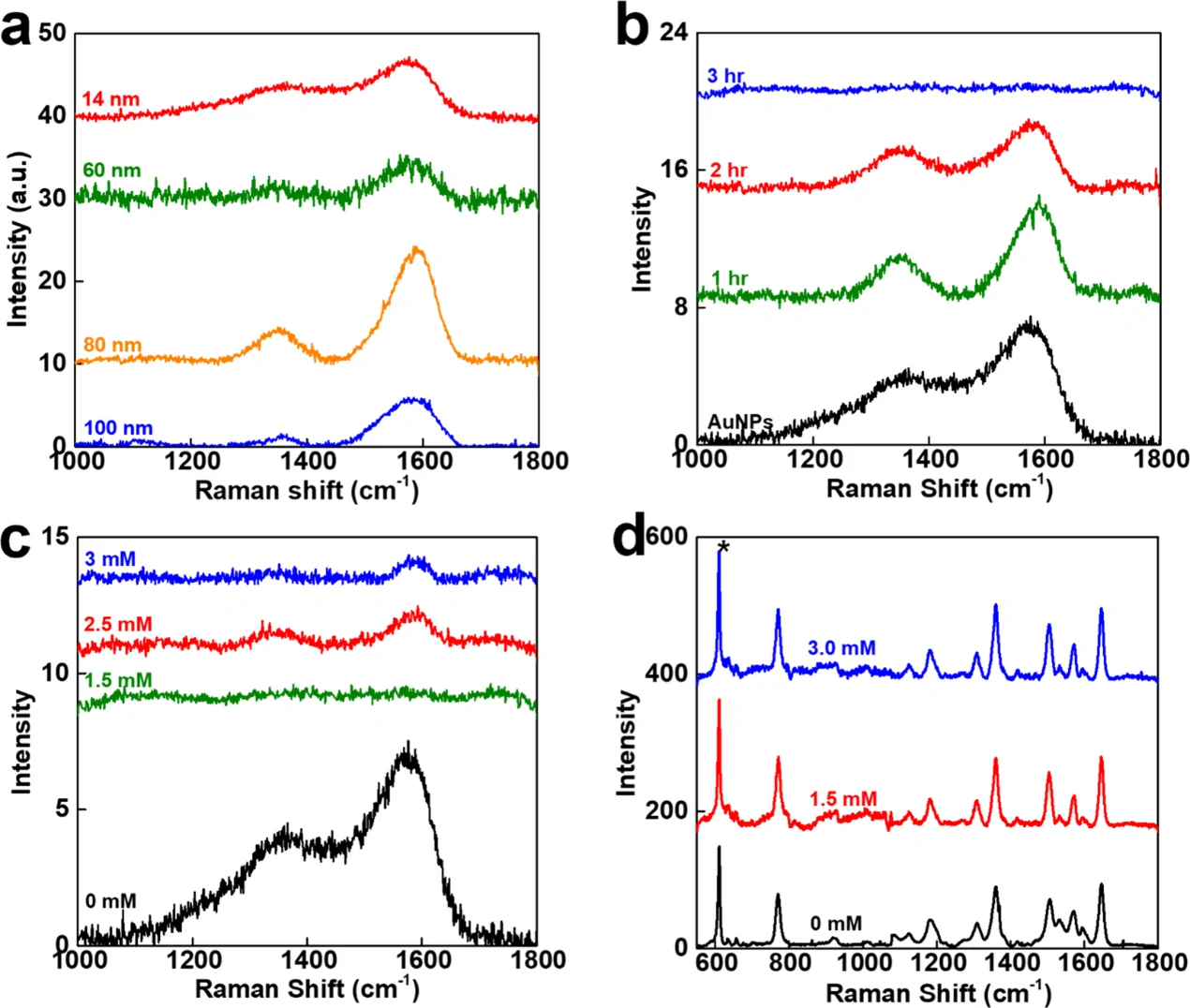
Raman background suppression and SERS enhancement via KBr surface treatment of AuNPs. (a) Raman spectra of AuNPs with four different sizes (14, 60, 80, and 100 nm) synthesized via microplasma reduction, all showing significant background signals in the fingerprint region (1000–1800 cm^–1^) that interfere with analyte detection. (b) Raman spectra of 14 nm AuNPs before and after treatment with 1.5 mM KBr for 3 h, showing substantial suppression of background features. (c) Magnified comparison of background regions before and after KBr treatment, confirming effective surface cleaning. (d) SERS spectra of 14 nm AuNPs with varying KBr concentrations (0, 1.5, and 3 mM) using rhodamine 6G (R6G) as a probe molecule. Optimal enhancement is observed at 1.5 mM KBr, while excessive Br^–^ results in signal suppression due to surface passivation.

To address this issue, 14 nm AuNPs were selected as a model to optimize surface cleaning via potassium bromide (KBr) treatment. As shown in Figure 3b,c, the addition of 1.5 mM KBr followed by gentle mixing for 3 h effectively suppressed background Raman features, indicating the successful displacement of adsorbed impurities and surface passivation. The impact of this surface purification on SERS activity was then assessed using R6G as a model analyte. Notably, treatment with 1.5 mM KBr led to an approximately 1.3-fold increase in the intensity of the R6G peak at 612 cm^–1^ compared to untreated AuNPs. However, increasing the KBr concentration further to 3 mM slightly reduced the signal (∼1.07-fold decrease from the 1.5 mM condition), as shown in Figure 3d. This trend can be explained by the dual role of the bromide ions. At moderate concentrations, Br^–^ ions act as effective competitive adsorbates that displace residual organic species, exposing cleaner Au surfaces and enhancing analyte–substrate interactions. However, at higher concentrations, excessive bromide coverage may lead to surface overpassivation or electrostatic repulsion effects, which hinder analyte adsorption and thus reduce the SERS enhancement. These findings confirm that halide-assisted surface engineering offers an efficient and tunable approach to minimize the Raman background and optimize the surface activity, which is critical for achieving background-free high-performance SERS substrates.

To further elucidate the role of KBr in modulating the surface properties and colloidal stability, zeta potential measurements were conducted on the AuNPs before and after halide treatment (Figure S5). All KBr-treated AuNP samples exhibited significantly more negative zeta potentials, indicating an increased surface charge density and improved electrostatic stabilization. This shift is consistent with previous studies showing that bromide ions can effectively displace surface-bound citrate molecules that are weakly adsorbed through electrostatic interactions.^30,31^ The ligand exchange mechanism between citrate and bromide was further verified by FT-IR spectroscopy (Figure S6). The FT-IR spectrum of citrate-stabilized AuNPs exhibited two characteristic carboxylate stretching vibrations at 1589 cm^–1^ (ν_as­(COO^–^)) and 1384 cm^–1^ (ν_s­(COO^–^)), corresponding to the asymmetric and symmetric modes of surface-adsorbed citrate groups. A weaker band at 1419 cm^–1^ indicated that a portion of the COO^–^ groups was coordinated to the AuNP surface through electrostatic interaction. After KBr treatment, this 1419 cm^–1^ band gradually disappeared within 3 h, demonstrating that Br^–^ ions effectively displaced citrate ligands on the gold surface.^32^ XPS analysis was performed to investigate the surface chemical composition of the 14 nm AuNPs after KBr treatment for different reaction times (0 to 3 h). As shown in Figures S8 and S9 and Tables S1–S3, the high-resolution spectra reveal clear binding energy peaks corresponding to the surface species. The C 1s and O 1s spectra indicate the presence of citrate residues on the initial gold surface.^31–34^ Specifically, the O 1s spectra display multiple components located at 529.5, 531.2 to 531.5, 532.4, and 535.7 eV, which are assigned to O–Au, O=C, C–OH, and condensed water, respectively.^35–38^ The initial Au 4f spectra exhibit typical metallic gold peaks at 82.9 and 86.6 eV,^39^ along with components at 83.4 and 87.1 eV.^40,41^ After the KBr addition, the Br 3d signal clearly appears with binding energies located at 68.5 eV (Br 3d_5/2_) and 69.5 eV (Br 3d_3/2_), verifying the successful adsorption of bromide ions.^42^ To quantitatively track the ligand displacement, the atomic percentages of these elements were evaluated across different treatment times. The atomic percentage of oxygen decreased from 43.61% at 0 h to 28.86% at 3 h, proving the removal of oxygen-rich citrate ligands. The Br atomic percentage increased from 0 to 1.36% at 2 h and stabilized at 0.95% at 3 h. The detectable Au atomic percentage increased from 0.31 to 0.88% over the same period. These ratio changes confirm a controlled adsorption of bromide ions. The treatment displaces bulky citrate molecules to expose active gold sites and successfully avoids excessive bromide accumulation that causes surface passivation. Importantly, this quantitative XPS data directly correlates with our Raman background suppression results. The significant reduction of citrate ligands perfectly explains the elimination of the broad background signals in the 1000 to 1800 cm^–1^ fingerprint region observed earlier. Furthermore, the high-resolution Au 4f spectra reveal a positive shift in binding energy for the 3 h KBr-treated sample. The Au 4f_7/2_ and Au 4f_5/2_ peaks shifted toward higher binding energies, reaching 84.3 and 88.0 eV at 3 h.^43^ This shift indicates an altered electronic environment on the gold surface. Highly electronegative bromide ions chemisorb onto the Au surface and withdraw electron density from the gold atoms. This electronic interaction stabilizes the cleaned active sites and modulates the local electronic structure. The altered structure actively facilitates ground-state charge transfer (ground-state CT) processes. This halide-induced electronic modulation acts synergistically with the physically exposed plasmonic hotspots. It provides an additional chemical enhancement mechanism to maximize the overall SERS performance.^44^ The replacement of citrate by Br^–^ not only purifies the nanoparticle surface but also enhances the electrostatic repulsion between particles, thereby reducing aggregation and promoting colloidal stability. The improved surface uniformity is beneficial for SERS applications. To evaluate colloidal stability after KBr treatment, the optical and hydrodynamic properties of AuNPs were monitored over 7 days. The UV–vis spectra showed a stable LSPR peak (554–561 nm, Figure S10), indicating consistent optical properties. Zeta potential remained highly negative (−75.9 to – 58.4 mV, Figure S11a), ensuring sufficient electrostatic stabilization.^45,46^ DLS results revealed only minor size fluctuations (100–160 nm) without the appearance of large aggregates (Figure S11b), which was further confirmed by SEM observations. These results demonstrate that the AuNPs maintain good colloidal stability for reliable SERS applications. A more homogeneous and cleaner surface enhances the accessibility and binding affinity of the analyte molecules, which in turn contributes to stronger and more reproducible Raman signals. The observed increase in the R6G signal intensity following KBr treatment aligns with this mechanism, further validating the role of halide ions in optimizing the physicochemical environment of the AuNP substrate for SERS. Based on this evidence, 1.5 mM KBr was identified as the optimal treatment concentration. It offers a balance between effective surface cleaning and preservation of analyte adsorption sites, enabling background suppression without compromising SERS sensitivity. Therefore, this optimized condition was employed in subsequent experiments for sensitive detection of environmental contaminants.

Halide ions, particularly bromide (Br^–^), are known to adsorb strongly onto noble metal surfaces such as gold through chemisorption mechanisms. Br^–^ was selected over Cl^–^ due to its stronger adsorption on Au surfaces, which enables the efficient displacement of residual citrate ligands while maintaining colloidal stability. Conversely, I^–^ was avoided because it binds too strongly, often inducing unfavorable nanoparticle aggregation and plasmon damping that are detrimental to SERS performance.^30,47–49^ This surface affinity allows Br^–^ to displace weakly bound organic stabilizers or residual byproducts, such as citrate or partially oxidized species, that are commonly introduced during nanoparticle synthesis. By competitively replacing these surface impurities, Br^–^ effectively ″cleans″ the nanoparticle surface, exposing a more uniform and chemically accessible gold interface. Specifically, Br^–^ ions occupy the same binding sites previously held by citrate. In this context, ″purification″ refers to clearing the spectroscopic background rather than creating a completely bare surface.^30,31^ This displacement process enhances the colloidal and surface electrostatic properties of the AuNPs, as reflected by the increased zeta potentials and reduced aggregation tendencies. More importantly, the removal of organic residues eliminates unwanted vibrational signals especially in the critical fingerprint region (1000–1800 cm^–1^) that often interfere with SERS detection of target molecules. Crucially, Br^–^ itself is Raman-inactive under typical excitation wavelengths and does not exhibit any characteristic Raman bands in the spectral range relevant to most SERS analyses. This is because of its simple monatomic ionic structure and the lack of polarizable chemical bonds that would produce significant Raman scattering, meaning it effectively creates a clean, Raman-silent baseline for accurate measurements, a mechanism successfully utilized in other high-performance SERS studies.^47–49^ As a result, Br^–^ treatment not only enhances SERS performance by improving analyte binding and field enhancement but also contributes no additional background, enabling clean, high-contrast Raman spectra. This property makes Br^–^ an ideal additive for engineering low-background, high-sensitivity SERS substrates, particularly in label-free and trace-level detection scenarios, where spectral clarity is paramount.

### 2.3 SERS Study of Plasma–Halide-Engineered AuNPs

To systematically evaluate and optimize the effect of the KBr treatment on the SERS performance of AuNPs, two representative environmental contaminants, R6G and MG, were selected as model analytes. SERS spectra were acquired using four different AuNP sizes (14, 60, 80, and 100 nm), as shown in Figure 4a. The characteristic Raman peaks of R6G located at 612, 772, 1182, 1360, 1506, and 1647 cm^–1^ were clearly identified and were consistent with the literature assignments. These peaks correspond to key vibrational modes: the 612 cm^–1^ peak arises from in-plane bending of C–C–C bonds; the 772 and 1182 cm^–1^ peaks are attributed to out-of-plane and in-plane C–H vibrations, respectively; while the peaks at 1360, 1506, and 1647 cm^–1^ reflect aromatic C–C stretching modes.^50–52^ Among the different particle sizes tested, the 100 nm AuNPs exhibited the highest SERS intensity, followed by 80, 60, and 14 nm particles (Figure 4b). This size-dependent trend is attributed to the localized surface plasmon resonance (LSPR) effect: Larger nanoparticles support stronger and more spatially extended electromagnetic fields upon light excitation, resulting in enhanced local field strength and greater Raman signal amplification. Moreover, larger AuNPs provide more favorable hotspot conditions, which further contribute to increased enhancement.^53^

**Figure 4:**
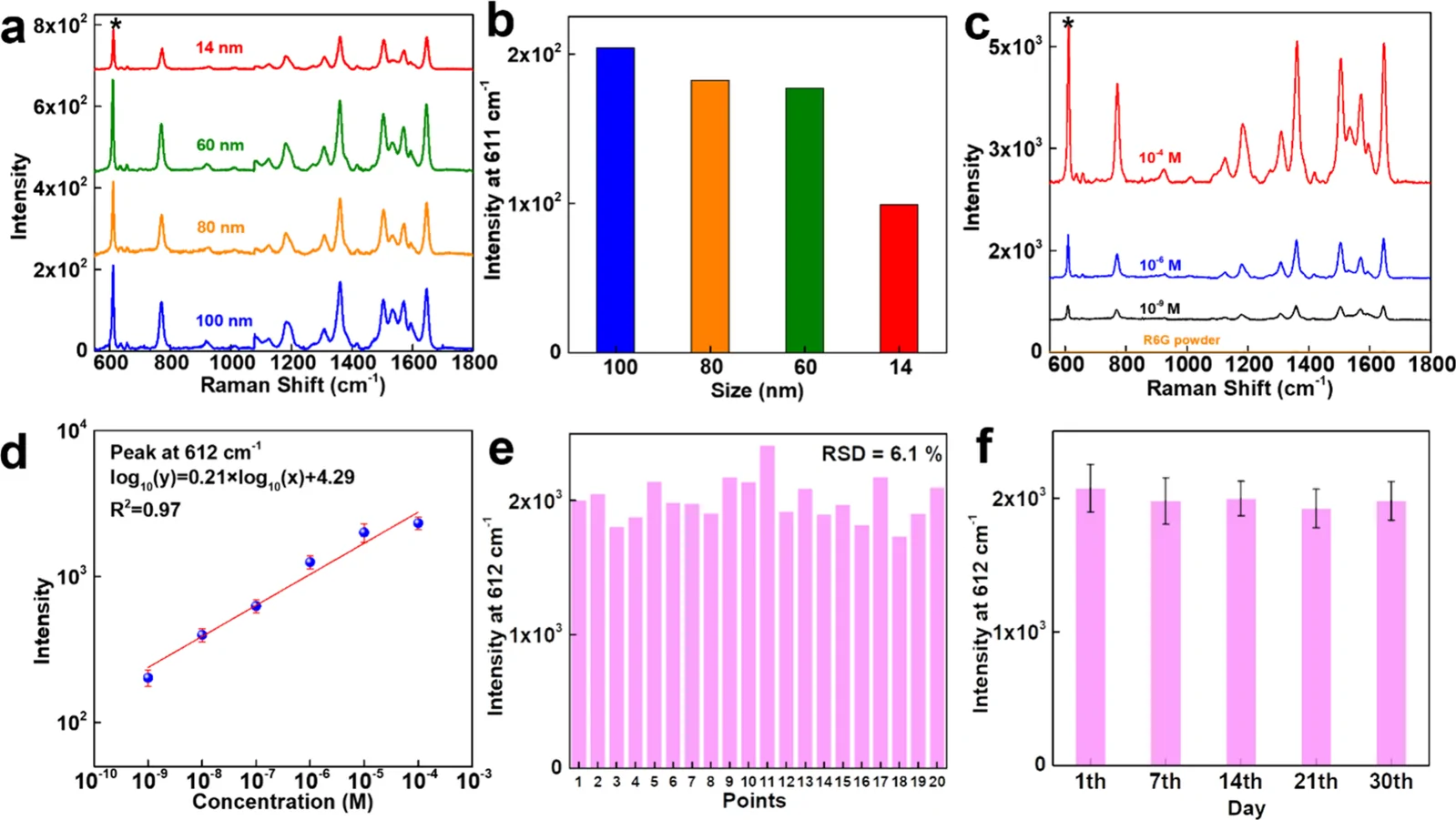
Optimization and evaluation of microplasma-synthesized, size-controlled AuNPs for R6G SERS detection. (a) Raman spectra of R6G obtained using AuNPs of different sizes (14, 60, 80, and 100 nm). (b) Comparison of Raman peak intensity at 612 cm^–1^ using different-sized AuNPs. (c) Raman spectra of R6G at concentrations from 10^–9^ to 10^–4^ M using 100 nm AuNPs. (d) Linear relationship of R6G SERS intensity (612 cm^–1^) with 100 nm AuNPs as a function of R6G concentration from 10^–9^ to 10^–4^ M. (e) Uniformity test: R6G SERS intensity (612 cm^–1^) with 100 nm AuNPs at random locations. (f) Time-stability study: R6G SERS intensity (612 cm^–1^) with AuNPs at different days.

To determine the detection limit of the optimized substrate, SERS measurements were performed for R6G at varying concentrations using 100 nm AuNPs. As shown in Figure 4c and Figure S12a, the system achieved a limit of detection (LOD) of 10^–9^ M. A strong linear relationship was observed between the SERS intensity at 612 cm^–1^ and R6G concentration across the range of 10^–9^ to 10^–4^ M (Figure 4d), highlighting the potential of the platform for quantitative detection. The SERS enhancement factor (EF) was calculated using the equation EF = (*I*_SERS_/*I*_Raman_) × (*C*_Raman_/*C*_SERS_), where *I*_SERS_ and *I*_Raman_ are the Raman intensities of R6G at 612 cm^–1^ with and without the SERS substrate, respectively, and *C*_SERS_ and *C*_Raman_ represent the analyte concentrations used in each case.^54^ The calculated EF was 2.12 × 10^6^, indicating a high enhancement efficiency resulting from the synergistic effects of halide-mediated surface cleaning and LSPR optimization. Control experiments were conducted to distinguish the individual contributions of plasma treatment and KBr modification. Under identical conditions, plasma-treated AuNPs exhibited significantly stronger SERS responses toward R6G than KBr-only samples (Figures S13–S15). Quantitative analysis using relative enhancement factor (REF) (at 10^–4^ M) shows higher enhancement for plasma-treated particles (7.90) compared to KBr-only samples (4.96).^55^ These results indicate that plasma treatment plays a dominant role, while KBr alone is insufficient, and their combination leads to improved overall SERS performance. In addition to the high sensitivity and background suppression, the practical deployment of SERS substrates requires excellent signal reproducibility and long-term stability. To evaluate reproducibility, we collected SERS spectra at 10 randomly selected positions on the KBr-treated 100 nm AuNP substrate using rhodamine 6G as a probe. The resulting relative standard deviation (RSD) was 6.1%, indicating a highly uniform signal distribution across the substrate surface (Figure 4e and Figure S12b). Furthermore, stability tests showed that the SERS intensity remained nearly unchanged after 30 days of storage under ambient conditions, with less than 7% signal degradation (Figure 4f and Figure S12h). These results demonstrate that our microplasma–halide-engineered AuNPs offer not only strong enhancement and low background but also consistent and durable performance. Such reproducibility and stability are critical for real-world applications, particularly in clinical diagnostics, environmental monitoring, and portable sensing platforms, where batch-to-batch consistency and a long shelf life are essential.

### Label-Free SERS Detection Using Plasma–Halide-Engineered AuNPs

To further validate the SERS performance of the AuNP substrates, MG was used as an additional target analyte. MG is a toxic synthetic dye widely used in aquaculture and industry; however, its carcinogenic and bioaccumulative properties have led to regulatory bans.^56^ Despite these restrictions, MG is still illicitly used, necessitating the use of sensitive, rapid, and reliable detection methods. Conventional techniques, such as HPLC and mass spectrometry, are accurate but require complex procedures. SERS offers a powerful alternative that enables label-free ultratrace detection with minimal sample preparation.^57^ Therefore, the development of low-background, high-sensitivity SERS substrates, as demonstrated in this study, is critical for the effective environmental monitoring and food safety surveillance of MG contamination. The SERS signals obtained from the KBr-treated and untreated AuNPs are compared in Figure 5a and Figure 5b, respectively. MG exhibits a characteristic Raman peak at 1615 cm^–1^, which is consistent with previous reports.^58,59^ As shown, the KBr-treated AuNPs yielded significantly stronger signals, with the 100 nm samples exhibiting a 1.95-fold increase in SERS intensity relative to untreated AuNPs. This enhancement is attributed to effective background suppression by Br^–^ ions, which exposes cleaner AuNP surfaces and improves analyte binding, thereby enhancing the SERS sensitivity. Using the optimized 100 nm KBr-treated AuNPs, the limit of detection (LOD) for MG was determined to be as low as 10^–10^ M (Figure 5c and Figure S18a). A strong linear correlation between the SERS signal at 1615 cm^–1^ and MG concentration was observed across the range of 10^–10^ to 10^–4^ M (Figure 5d). The enhancement factor (EF), calculated using the same formula as previously described, was 2.66 × 10^6^, demonstrating the strong signal-amplifying capability of the background-free substrates.

**Figure 5:**
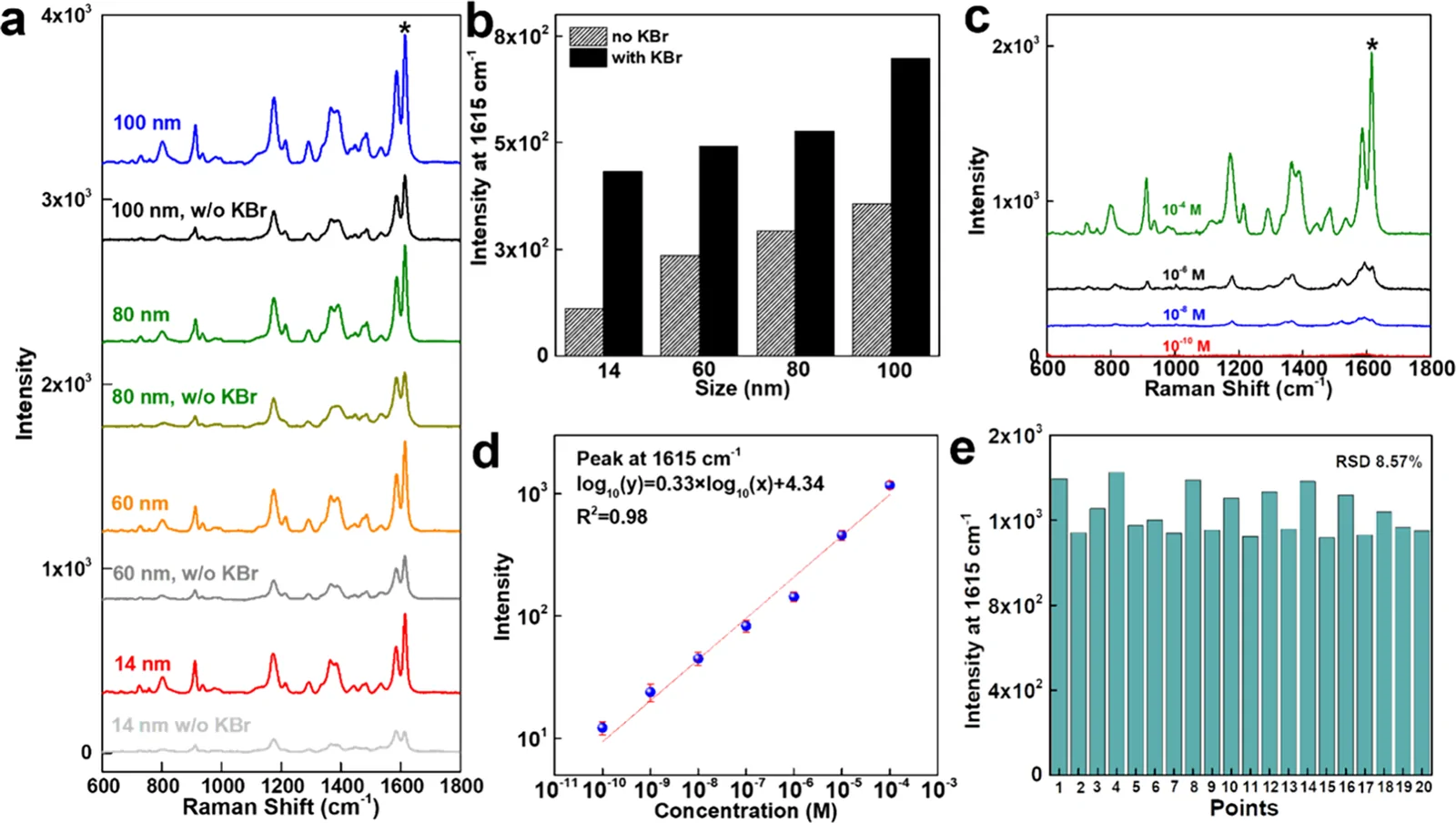
SERS evaluation of size-controlled AuNPs synthesized by microplasma and KBr treatment. (a) Raman spectra of MG obtained using size-controlled AuNPs (14, 60, 80, and 100 nm) synthesized via microplasma, comparing samples with and without KBr treatment. (b) Quantitative comparison of SERS intensities at 1615 cm^–1^ for MG on AuNPs of different sizes, highlighting the enhancement effect of KBr treatment. (c) Raman spectra of MG at concentrations from 10^–10^ to 10^–4^ M using 100 nm AuNPs. (d) Linear relationship between SERS intensity at 1615 cm^–1^ and MG concentration (10^–10^ to 10^–4^ M) using 100 nm AuNPs. (e) Uniformity test: SERS intensity of MG (1615 cm^–1^) using 100 nm AuNPs at randomly selected spots.

To assess the signal uniformity, SERS spectra were collected from 20 random locations on the KBr-treated substrate, yielding a relative standard deviation (RSD) of 8.57% (Figure 5e and Figure S18b), confirming good reproducibility. To further investigate the influence of KBr treatment on analyte adsorption, UV–vis spectroscopy was performed using 100 nm AuNPs mixed with either R6G or MG solutions. Compared to the untreated samples, KBr-treated AuNPs showed more pronounced decreases in absorbance at 526 nm (R6G) and 615 nm (MG) (Figure S19), indicating enhanced molecular adsorption. This result supports the hypothesis that Br^–^ ions improve surface cleanliness and increase the adsorption efficiency of target analytes, contributing to stronger SERS signals. Compared to previous reports, which often rely on the multistep fabrication of complex nanohybrids or composite substrates to achieve comparable sensitivity (Table S8), our approach offers a simple, scalable, and effective alternative. Notably, representative systems such as AuNPs@MIL-100­(Fe) and AuNPs/TiO_2_/Ti_3_C_2_ heterostructures reported LODs of 3.04 × 10^–10^ and 2.71 × 10^–10^ M for MG detection, respectively, while Au@Ag nanorod-based substrates typically achieved detection limits in the 10^–9^ M range (Table S8). In comparison, the present plasma–halide-engineered AuNPs achieve a comparable LOD of 10^–10^ M using a relatively straightforward preparation strategy. These findings highlight the potential of halide-treated low-background AuNPs as practical and efficient SERS platforms for trace-level detection without requiring additional structural modifications.

The enhanced Raman scattering observed is suggested to be primarily driven by two synergistic factors: (1) the plasmonic field intensification associated with size-optimized AuNPs and (2) the surface purification effect induced by Br^–^ treatment. Notably, the 1615 cm^–1^ Raman mode of MG, corresponding to the aromatic C=C stretching vibration, showed the most significant enhancement, suggesting the selective amplification of vibrational modes with strong polarizability and spatial alignment near the hotspot regions. The effective removal of citrate by Br^–^ not only minimized spectral interference but also increased the surface availability for MG adsorption, especially through π–π stacking and electrostatic interactions. Furthermore, the field enhancement near the nanoparticle surface likely couples more efficiently with symmetric aromatic stretching modes such as 1615 cm^–1^, which are more polarizable and surface-oriented, thereby contributing to the observed selectivity. This mechanism reinforces the importance of surface cleanliness and nanogap accessibility for enabling both strong and selective SERS responses, with implications for broader molecular sensing applications.

### 2.5 Surface Charge Effect on SERS Selectivity

Adsorption is the critical step in SERS, and electrostatics strongly modulate adsorption at metal–solution interfaces. After KBr treatment, the AuNPs acquire a more negative surface potential, which could enhance colloidal stability yet disfavor uptake of coanionic analytes. We therefore asked whether background suppression alone (via Raman-inactive Br^–^ cleaning) improves detection, or whether electrostatic repulsion negates the gain. To probe this, we selected two anionic molecules, methyl red (MR) and folic acid (FA),^60–62^ that are negatively charged under our measurement pH and thus constitute a test case for adsorption onto negatively charged AuNPs. As shown in Figure S20, MR exhibits a characteristic Raman peak at 1387 cm^–1^, consistent with the previous report.^63^ The lowest detectable concentration of MR was determined to be 10^–6^ M, and a strong linear correlation between the SERS intensity at 1387 cm^–1^ and MR concentration was observed from 10^–6^ to 10^–4^ M. For FA detection using 100 nm AuNPs, a dominant Raman band was observed at 680 cm^–1^, which shows a slight shift compared with the powder spectrum (Figure S21b), likely due to the interaction between folic acid molecules and the Au surface, leading to changes in the local chemical environment and adsorption configuration. A detection limit of 5 × 10^–4^ M was achieved, and a clear linear relationship was obtained between the SERS intensity at 680 cm^–1^ and FA concentration ranging from 5 × 10^–4^ to 10^–3^ M (Figure S22a). Compared to the detection limits obtained for R6G and MG, both MR and FA exhibited higher detection limits, indicating that the AuNP substrates display analyte selectivity. Even though the Raman background of the substrate was effectively reduced through the halide-assisted surface cleaning process, the detection sensitivity remained strongly influenced by the electrostatic interactions between the analyte and the SERS-active surface. These findings suggest that while surface purification significantly improves spectral clarity and baseline stability, achieving optimal SERS enhancement still requires electrostatic complementarity between the analyte and the clean plasmonic surface. This highlights that both surface cleanliness and charge compatibility play synergistic roles in determining the overall SERS performance.

## 3 Conclusions

In summary, we demonstrated a simple, green, and scalable strategy to deliver background-free, label-free, and reproducible SERS using plasma–halide-engineered gold nanoparticles. Ambient microplasma enables size-tuned AuNPs (14–100 nm), while Raman-inactive Br^–^ competitively displaces citrate, suppressing baseline features in the 1000–1800 cm^–1^ fingerprint window without introducing new peaks. An optimal window (∼1.5 mM, ∼3 h) cleans the surface yet avoids passivation, preserving plasmonic response and improving adsorption. The resulting substrates achieve EF >10^6^, subnanomolar LODs (10^–9^ M R6G; 10^–10^ M MG), broad linear ranges, low spot-to-spot variability (RSD <∼8%), and week-scale stability (>90% signal retention), underscoring measurement fidelity and practical robustness. By decoupling surface cleaning from plasmonic design, this approach eliminates the need for complex hybrids or harsh chemistries and establishes a reliable route to quantitative trace analysis, particularly for cationic species. We anticipate utility in environmental monitoring, food safety, and bioanalytical sensing, and see opportunities to extend the platform to diverse analytes and complex matrices, as well as to device-level implementations (e.g., paper/flexible substrates). Future work will refine surface–adsorbate energetics and hotspot uniformity with advanced mapping/spectroscopy, further strengthening the design rules for clean-surface, high-fidelity SERS.

## 4 Experimental Section

### Plasma Syntheses of AuNPs

To prepare the precursor solutions, 0.75 mM chloroauric acid (HAuCl_4_) was mixed with different concentrations of sodium citrate (2, 5, and 10 mM), which acted as both reducing and stabilizing agents. Each solution was subjected to a microplasma treatment for 20 min, as illustrated in Scheme 1. During this ambient microplasma process, an argon-discharged plasma generates a rich mixture of reactive species at the gas–liquid interface including hydrated electrons, ions, and radicals. These species efficiently drive the rapid reduction of Au^3+^ precursors and subsequent nanoparticle nucleation without the need for harsh reductants or elevated temperatures. Extensive details regarding the physical setup, electrode configuration, and fundamental mechanisms of the atmospheric-pressure microplasma system can be found in our prior foundational studies.^19–22^ During this process, argon gas was introduced into the system at a controlled flow rate of 25 sccm for the synthesis of AuNPs with average diameters of 100, 80, and 60 nm, depending on the citrate concentration. To synthesize smaller AuNPs with an average diameter of 14 nm, a higher argon flow rate of 40 sccm was used while maintaining a sodium citrate concentration of 10 mM. After plasma treatment, the resulting solutions were centrifuged at 9000 rpm for 20 min to remove unreacted precursors and byproducts. The purified AuNPs were redispersed in deionized (DI) water after 5 min of sonication to ensure uniform dispersion for subsequent characterization. The obtained AuNP colloidal solutions were stored at 4 °C in sealed glass bottles to minimize exposure to air and prevent potential surface oxidation. No additional stabilizing agents were added during storage. Under these conditions, the nanoparticles maintained good colloidal stability and preserved their plasmonic properties for subsequent SERS measurements.

### Characterization Methods

The optical properties of the synthesized AuNPs were analyzed using a JASCO V-676 UV–vis absorbance spectrophotometer. All measurements were carried out in a matched pair of 1 cm path-length quartz cuvettes, with the corresponding solvents used as baseline references to eliminate background contributions. SEM was employed to further examine the surface morphology and aggregation state of AuNPs. SEM images were acquired using a JEOL JSM-IT800 system, which offers high-resolution images. TEM analysis was conducted to examine the morphology and structural features of AuNPs. Imaging was performed using a Spectra 300 FEG-S/TEM instrument operating at an accelerating voltage of 300 kV. TEM specimens were prepared by dry-casting droplets of colloidal AuNP solution onto Formvar/carbon-coated copper grids (300 mesh, EM Resolutions Ltd.). The particle size distributions and lattice spacings were analyzed using Digital Micrograph version 3.7. In addition, the surface charge and colloidal stability of the AuNPs were evaluated via zeta potential measurements using an Otsuka ELSZ-2000 zeta potential and particle size analyzer. XPS measurements were performed using an ULVAC-PHI Quantes spectrometer equipped with a monochromatic Al Kα X-ray source (*h*ν = 1486.6 eV), with a pass energy of 150.0 eV and an analysis area ranging from 7.5 to 1400 μm. Both survey and high-resolution spectra were collected to analyze the surface chemical composition and bonding states of the samples.

### SERS Measurement

The synthesized AuNP colloidal solution was treated with KBr. The optimized condition for the Raman measurement is 1.5 mM KBr with stirring for 3 h. Prior to substrate preparation, the silicon wafers (0.5 cm × 0.5 cm) were sequentially cleaned by ultrasonication in ethanol, acetone, and ultrapure water for 5 min each and subsequently dried. Fifty microliters of synthesized AuNP colloidal solution was drop-cast onto a precleaned silicon wafer (0.5 cm × 0.5 cm) and subsequently dried on a hot plate at 50 °C. The dried substrate was then immersed in an R6G solution for 30 min to allow molecular adsorption. After incubation, the substrate was dried on a hot plate at 50 °C and subjected to Raman spectroscopy analysis. This initial procedure was used to identify the optimal AuNP size for the SERS enhancement. Following the optimization, a larger volume of AuNPs was used to prepare the final SERS substrates. To ensure a homogeneous film and minimize the coffee-ring effect, a sequential drop-casting strategy was employed. The AuNP colloidal solution (total 200 μL) was deposited onto a cleaned 0.5 cm × 0.5 cm silicon wafer in small increments of 25 μL. Each droplet was allowed to dry slowly at 50 °C before the subsequent addition. The AuNP colloidal solution (200 μL) was drop-cast onto a 0.5 cm × 0.5 cm silicon wafer and dried at 50 °C. The substrate was then sequentially immersed in an R6G solution for 30 min and in MG, MR, and FA solution for 1 h separately to ensure adequate molecular coverage. After the final adsorption step, the substrate was dried again at 50 °C before Raman measurements were conducted. All substrates were subjected to the exact same dye concentration, volume, and immersion time to ensure uniform adsorption. Because of this consistency, any differences in the resulting SERS signals are driven by the substrates’ intrinsic properties rather than local concentration variations. Micro-Raman spectroscopy was performed at room temperature using a JASCO NRS-5100 spectrometer equipped with a 532 nm laser excitation source. The laser power was carefully adjusted and maintained at 3.7 mW during all the measurements to prevent thermal damage to the samples. Prior to data acquisition, the spectrometer was calibrated using a silicon wafer, which exhibits a characteristic Raman peak at 520 cm^–1^. For the control experiments, 50 μL of the 14 nm gold nanoparticles treated only with plasma, as well as the AuNPs synthesized by the conventional reduction method treated only with KBr, were respectively deposited onto silicon wafers. All subsequent sample preparation steps and Raman measurement conditions were kept strictly identical to those used for the main experimental groups.

## Supplementary Material



## Notes

Detailed Raman spectra for multiple batches, UV–vis absorption spectra, FT-IR spectra, XPS spectra, zeta potential and DLS analysis over time, enhancement factor calculations, and additional experimental details and figures (PDF)
