# Supplementary material for: Background-Free, Label-Free, and Reproducible SERS via Plasma–Halide-Engineered Gold Nanoparticles
Source: ACS Meas Sci Au. 2026 May 4;6(4):937–48. doi: 10.1021/acsmeasuresciau.5c00196 (PMC13495453; doi:10.1021/acsmeasuresciau.5c00196)
Supplement: Supplementary file 1 [file tg5c00196_si_001.pdf]

Background-Free, Label-Free, and Reproducible SERS via Plasma–Halide-Engineered Gold Nanoparticles

(Supporting information)

Yen Ling Chiu[1], Ya Chi Chen[1], Loid Elizabeth Urtecho Navas[1] and Wei-Hung Chiang[1, 2]*

[1]Department of Chemical Engineering, National Taiwan University of Science and Technology, Taipei 10607, Taiwan

[2]Sustainable Electrochemical Energy Development (SEED) Center, National Taiwan University of Science and Technology, Taipei City 10607, Taiwan

* Corresponding author E-mail address: whchiang@mail.ntust.edu.tw (W.H.C.).

S1 Conventional Citrate Synthesized AuNPs for Control Experiments

To prepare AuNPs via the conventional citrate reduction method, 0.75 mM chloroauric acid ($HAuCl_4$) was mixed with 2 mM sodium citrate in deionized (DI) water, matching the precursor concentrations used for the plasma-synthesized 100 nm AuNPs. Sodium citrate acted as both the reducing and stabilizing agent. The reaction mixture was stirred at 500 rpm under room temperature conditions for 2 h. During the reaction, a gradual color change was observed, with the solution transitioning from an initial transparent appearance to a dark purple color, indicating the formation of AuNPs. After synthesis, the resulting colloidal solution was characterized by UV–Vis spectroscopy, followed by the addition of KBr to a final concentration of 1.5 mM. The mixture was then incubated for 3 h prior to subsequent Raman measurements.

S2 Calculation of the Analytical Enhancement Factor (AEF)

The SERS enhancement factor (EF) is evaluated as an Analytical Enhancement Factor (AEF), which is widely adopted in analytical SERS studies to compare practical and relative substrate performance under identical optical and adsorption conditions. The AEF for Rhodamine 6G (R6G) was estimated using the following formula:

$$EF = (I_{SERS} / I_{Raman}) \times (C_{Raman} / C_{SERS}) = (202.708 / 9.58) \times (10^{-4} / 10^{-9}) = 2.12 \times 10^{6}.$$

To determine the individual values, both the SERS intensity I_{SERS} and the normal Raman intensity I_{Raman} were determined based on statistical analysis from three independent batches. For each batch, spectra were collected from 20 random positions, and the average intensity at 612 cm^{-1} was used for calculation. The I_{SERS} values were obtained from KBr-treated AuNP substrates, while the I_{Raman} values were measured on a bare silicon substrate using 10^{-4} M R6G under identical experimental conditions. The corresponding spectra and statistical summaries are presented in Figure S16-S17 and Table S5.

S3 Relative SERS Intensity Comparison at the Same Analyte Concentration

To evaluate the relative SERS performance of different substrates under identical experimental conditions, an intensity-based comparison was adopted. According to previous reports[1], The relative enhancement factor (REF) between two different substrates was calculated by applying:

$$REF = (I_{Surf.A} N_{Surf.A})/(I_{Surf.B} N_{Surf.B})$$

where I represents the SERS intensity of the characteristic Raman band and N represents the number of molecules probed on each substrate. Under identical analyte concentration and measurement conditions, the number of probed molecules on different substrates can be reasonably assumed to be comparable. Therefore, the expression can be simplified as:

$$REF = I_{Surf.A} / I_{Surf.B}$$

where $I_{Surf.A}$ represents the Raman intensity obtained from the SERS-active substrate (including plasma-treated and KBr-only AuNPs), and $I_{Surf.B}$ represents the Raman intensity obtained from the reference sample (10^{-4} M) without a SERS-active substrate. All measurements were performed at the same analyte concentration and under identical experimental conditions to ensure a valid comparison.

Plasma-only

REF =75.72 / 9.58= 7.90

KBr only

REF = 47.55 / 9.58 = 4.96

Based on the averaged intensities, the REF of plasma-treated AuNPs and KBr-only samples were calculated to be 7.90 and 4.96, respectively. This result indicates that the plasma-treated substrates provide a significantly higher SERS enhancement compared to the KBr-only condition. The corresponding spectra and statistical summaries are presented in Figure S13-S15 and Table S6.

S4 Statistical Analysis of Inter-Batch Reproducibility

To further evaluate inter-batch variation, one-way analysis of variance (ANOVA) was performed using Raman intensities at 612 cm^{-1} obtained from three independently synthesized AuNP batches prepared on different days and using different plasma reactors under identical R6G conditions. Each batch included 20 randomly selected measurement points. The ANOVA results yielded an F value of 0.82 with a corresponding p value of 0.447 at a significant level of 0.05. Since the p value is greater than 0.05, no statistically significant difference was observed among the three batches. These results confirm that inter-batch variability is minimal and that the synthesis procedure exhibits good day-to-day reproducibility.

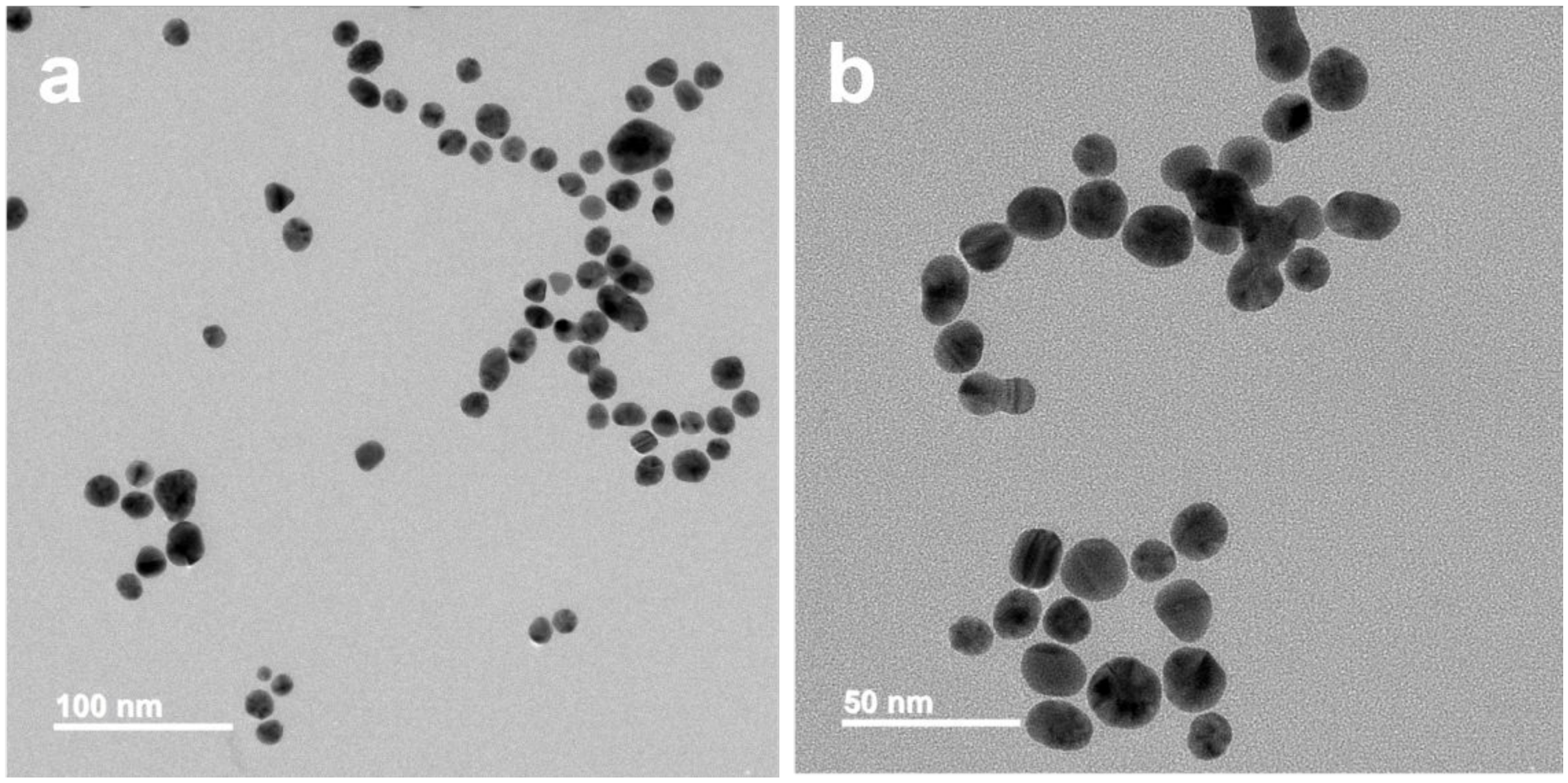

Figure S1 TEM images of the synthesized 14 nm AuNPs.

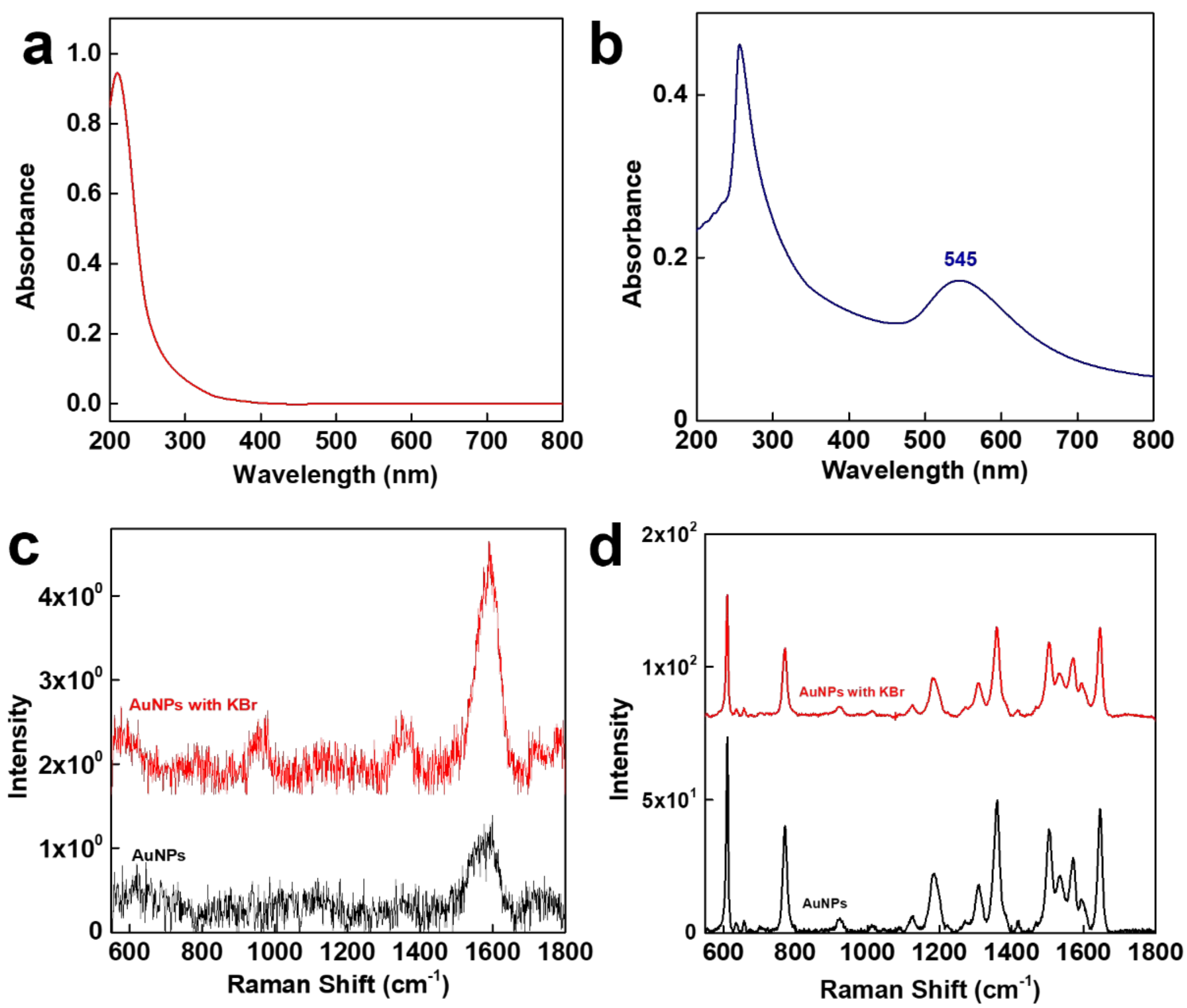

Figure S2 Characterization and SERS performance of AuNPs fabricated by the citrate reduction method.

(a, b) UV-Vis absorption spectra of the AuNPs reaction mixture after (a) 20 min and (b) 2 hours of reaction. (c) Background Raman spectra of the synthesized AuNPs. (d) SERS spectra of 10^{-4} M rhodamine 6G (R6G) enhanced by the AuNPs. The black and red lines in (c) and (d) represent the spectra obtained without and with the addition of KBr, respectively.

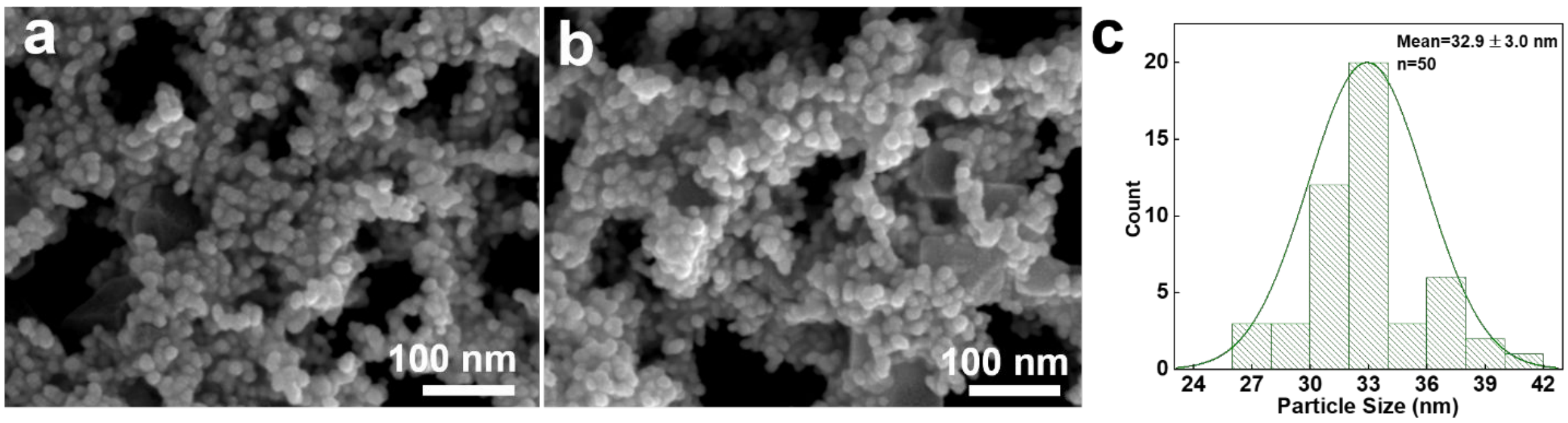

Figure S3 (a,b) SEM images of AuNPs synthesized via the conventional citrate reduction method. (c) Particle size distribution obtained from SEM analysis, showing an average particle diameter of ~32 nm. Scale bars: 100 nm.

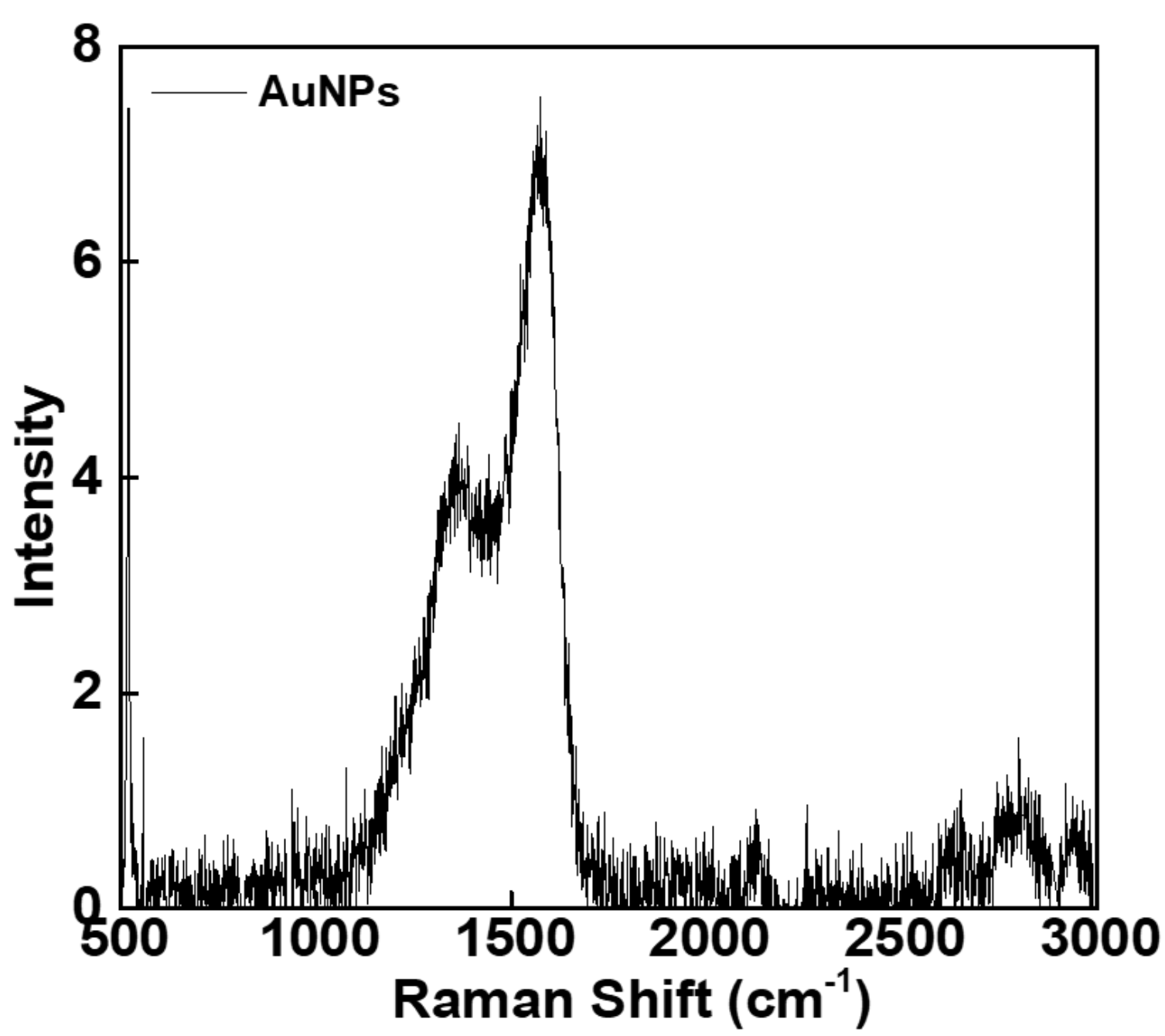

Figure S4 Raman spectrum of 14 nm AuNPs.

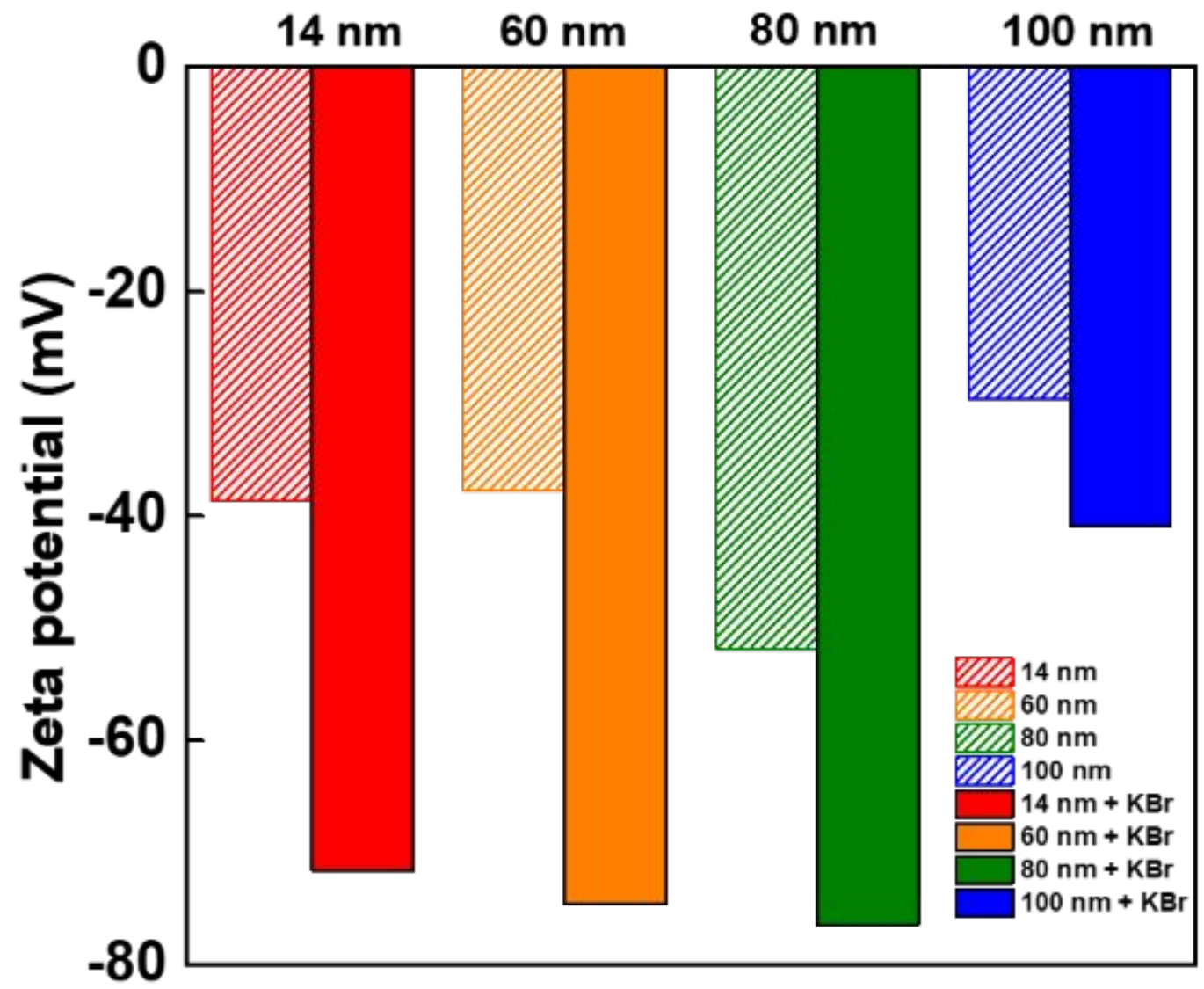

Figure S5 Zeta potential measurements of 14, 60, 80, and 100 nm AuNPs before and after KBr treatment.

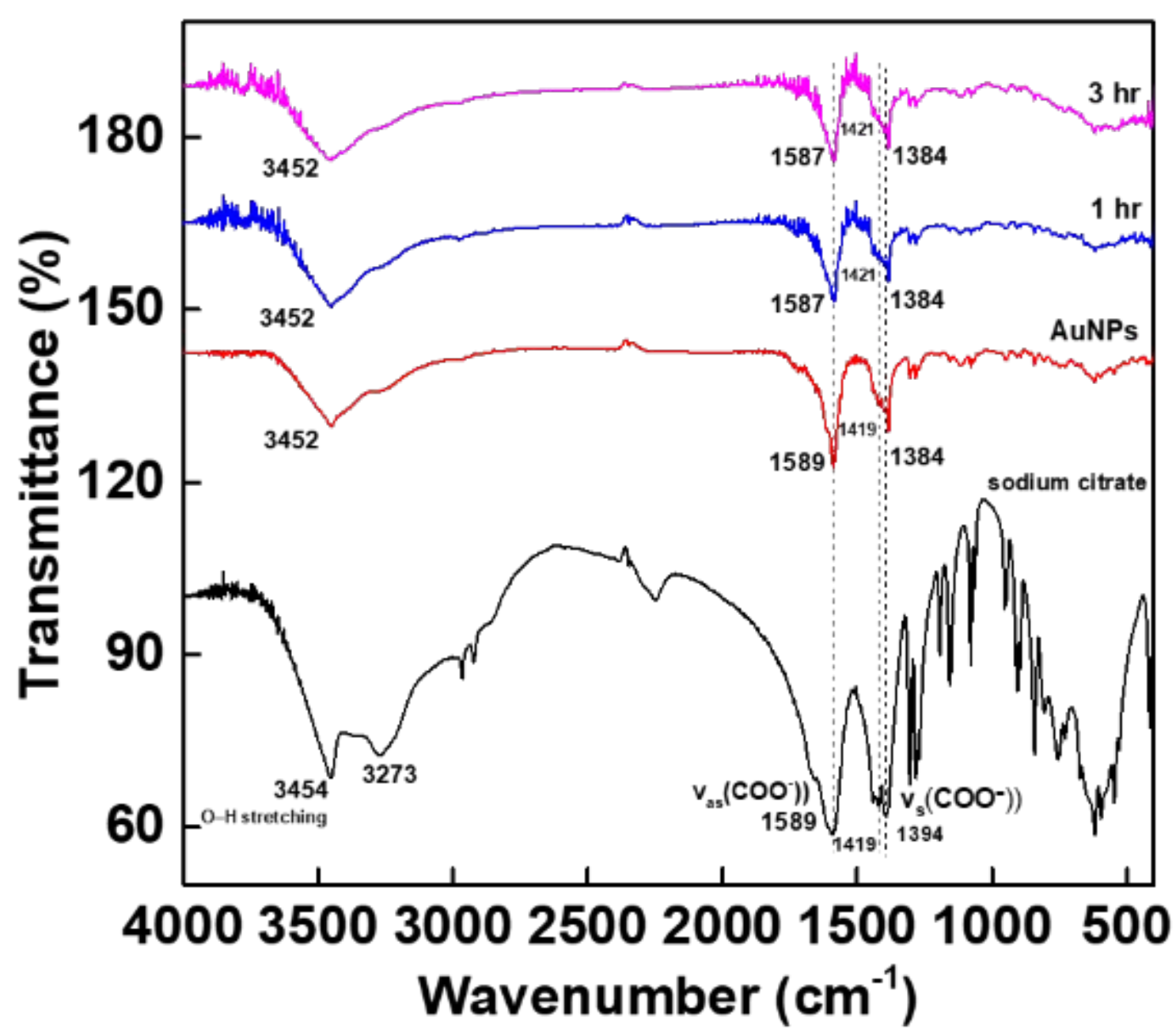

Figure S6 FT-IR spectra of sodium citrate, citrate-stabilized AuNPs, and KBr-treated AuNPs recorded at different treatment times (0, 1, and 3 h).

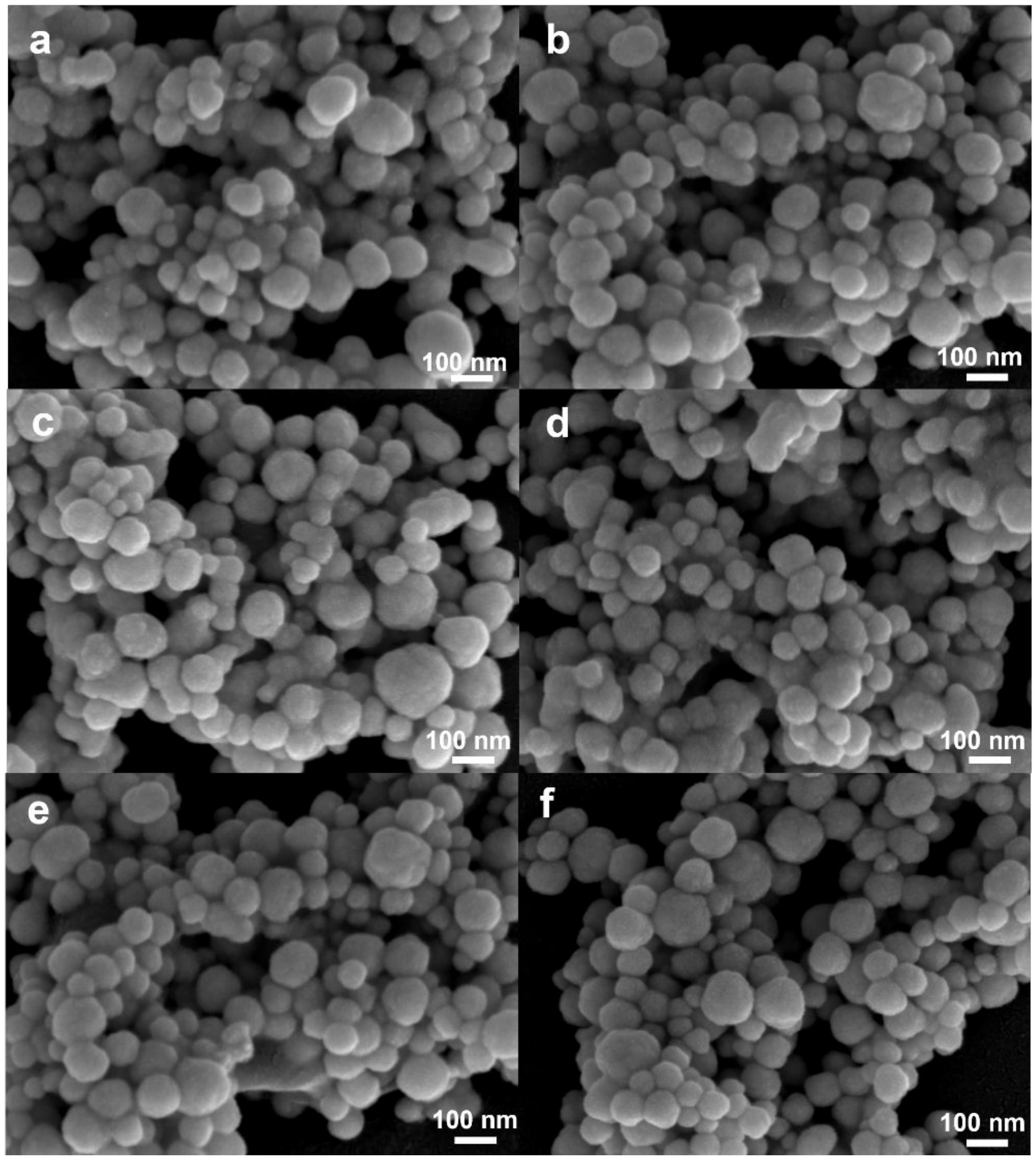

Figure S7 SEM images recorded from multiple regions (a–f) of the SERS substrate prepared by depositing 200 μL of 100 nm AuNPs on a silicon wafer, confirming the uniform surface morphology and consistent nanoparticle aggregation across the substrate. Scale bars: 100 nm.

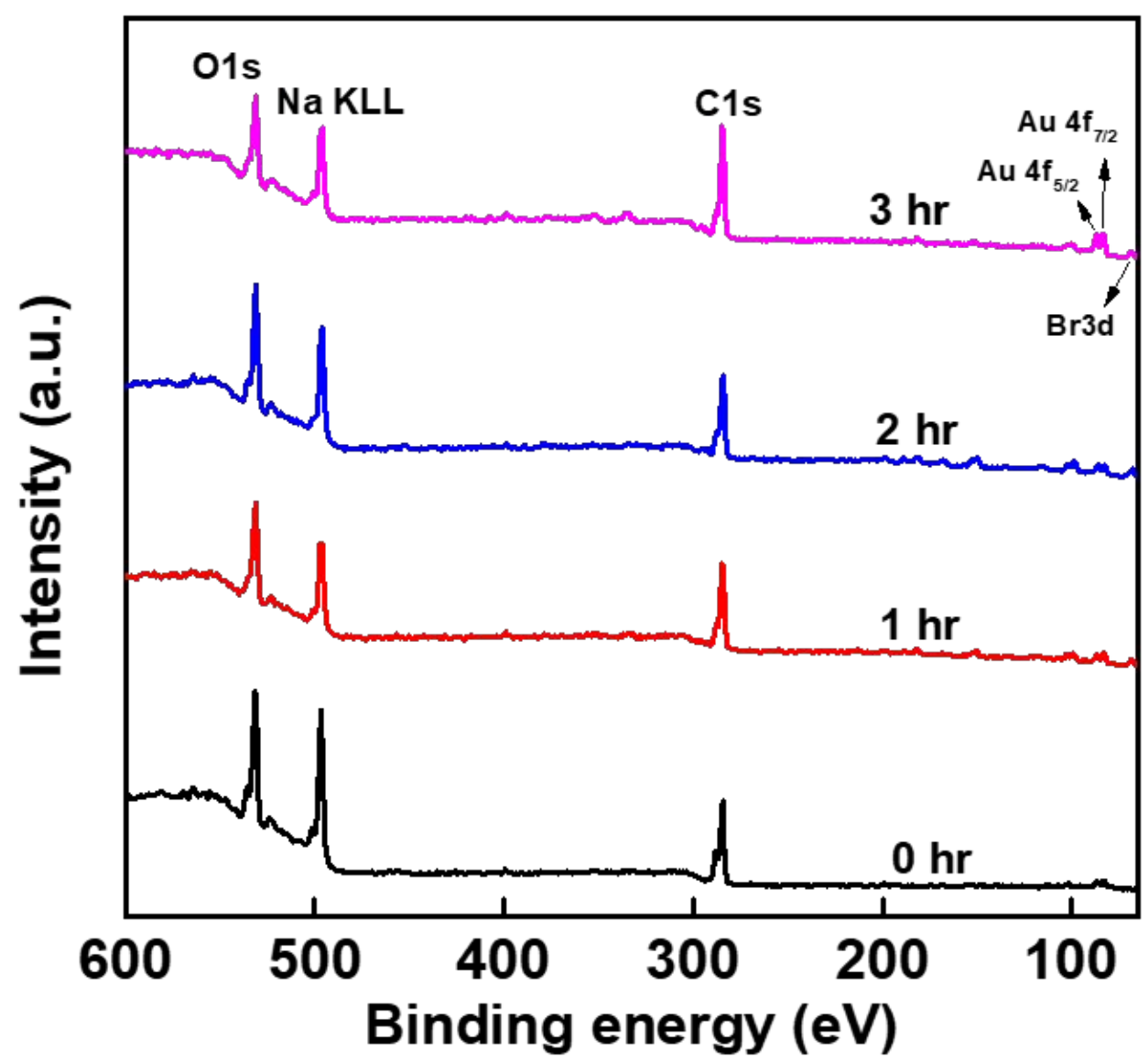

Figure S8 Full XPS survey spectra of the 14 nm AuNPs subjected to KBr treatment for 0, 1, 2, and 3 hours.

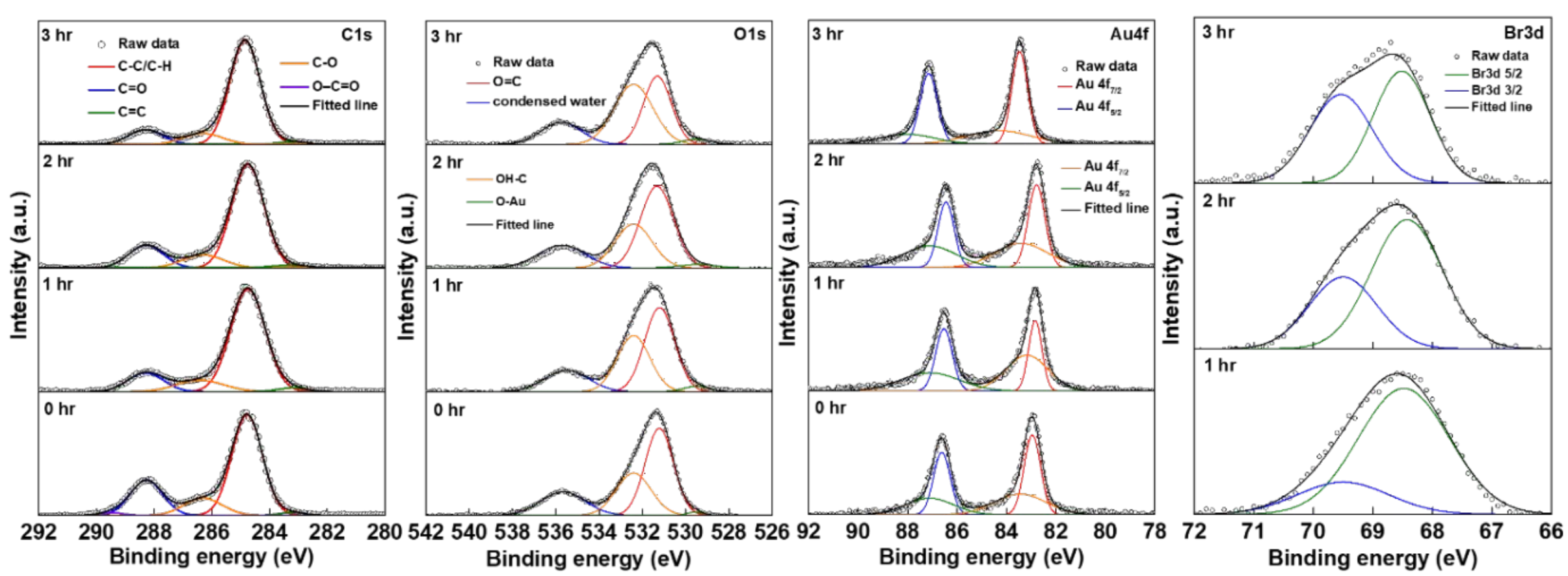

Figure S9 High-resolution XPS spectra of the C 1s, O 1s, Au 4f, and Br 3d core levels for the 14 nm AuNPs treated with KBr for 0, 1, 2, and 3 h.

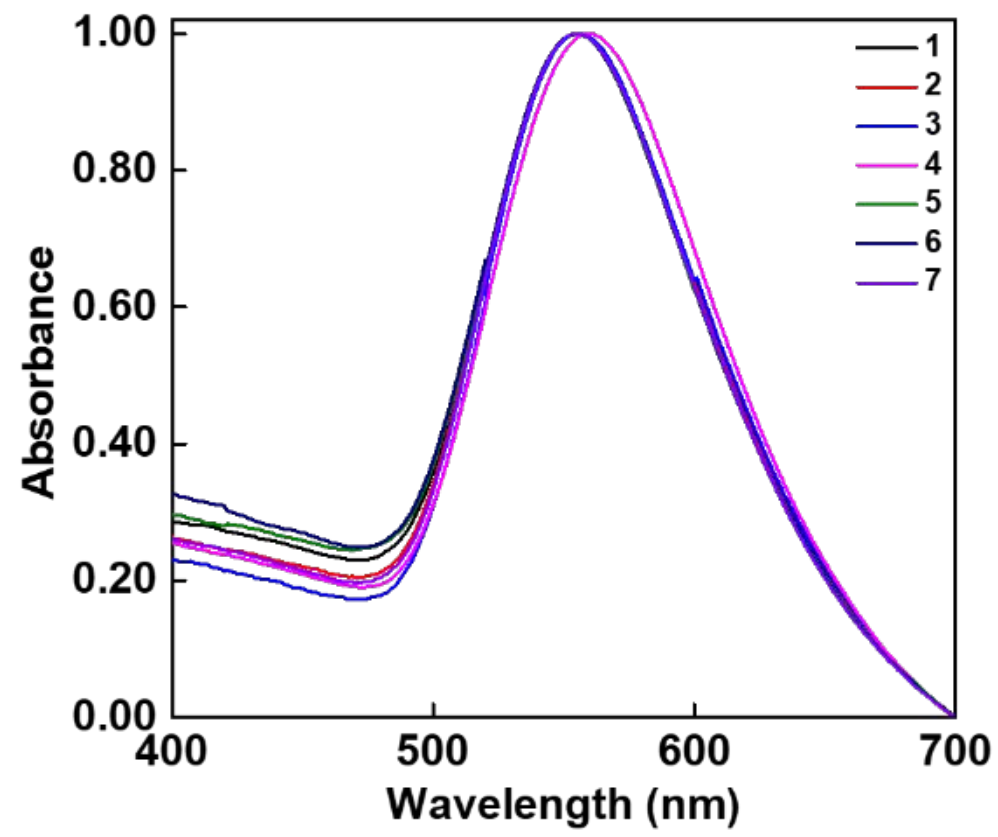

Figure S10 UV-Vis spectra of plasma-synthesized 100 nm gold nanoparticles treated with KBr monitored over 7 days.

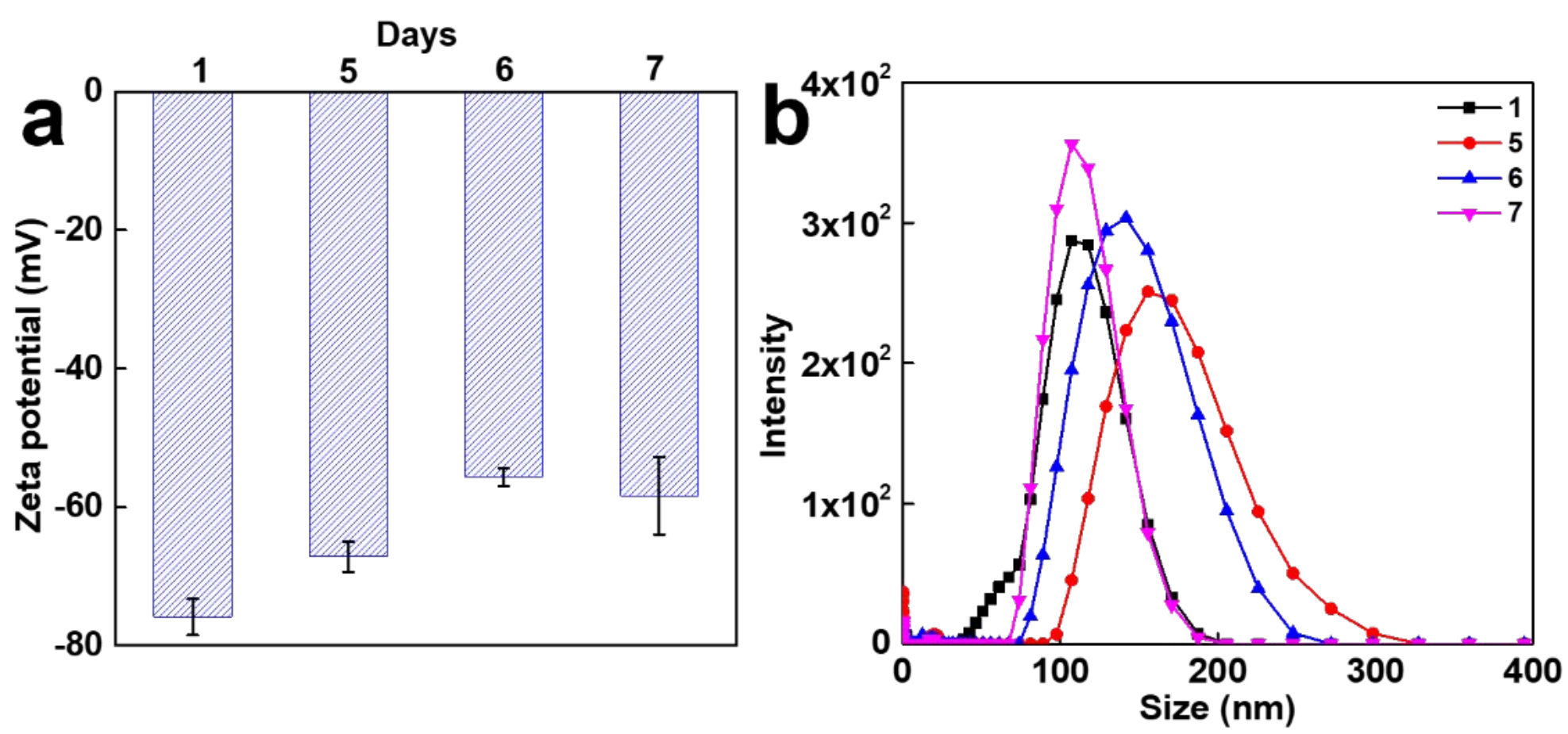

Figure S11 Time-dependent characterization of AuNPs measured at Day 1, Day 5, Day 6, and Day 7. (a) Zeta potential values of AuNPs at different days. (b) Intensity-weighted hydrodynamic size distributions of AuNPs obtained from DLS measurements.

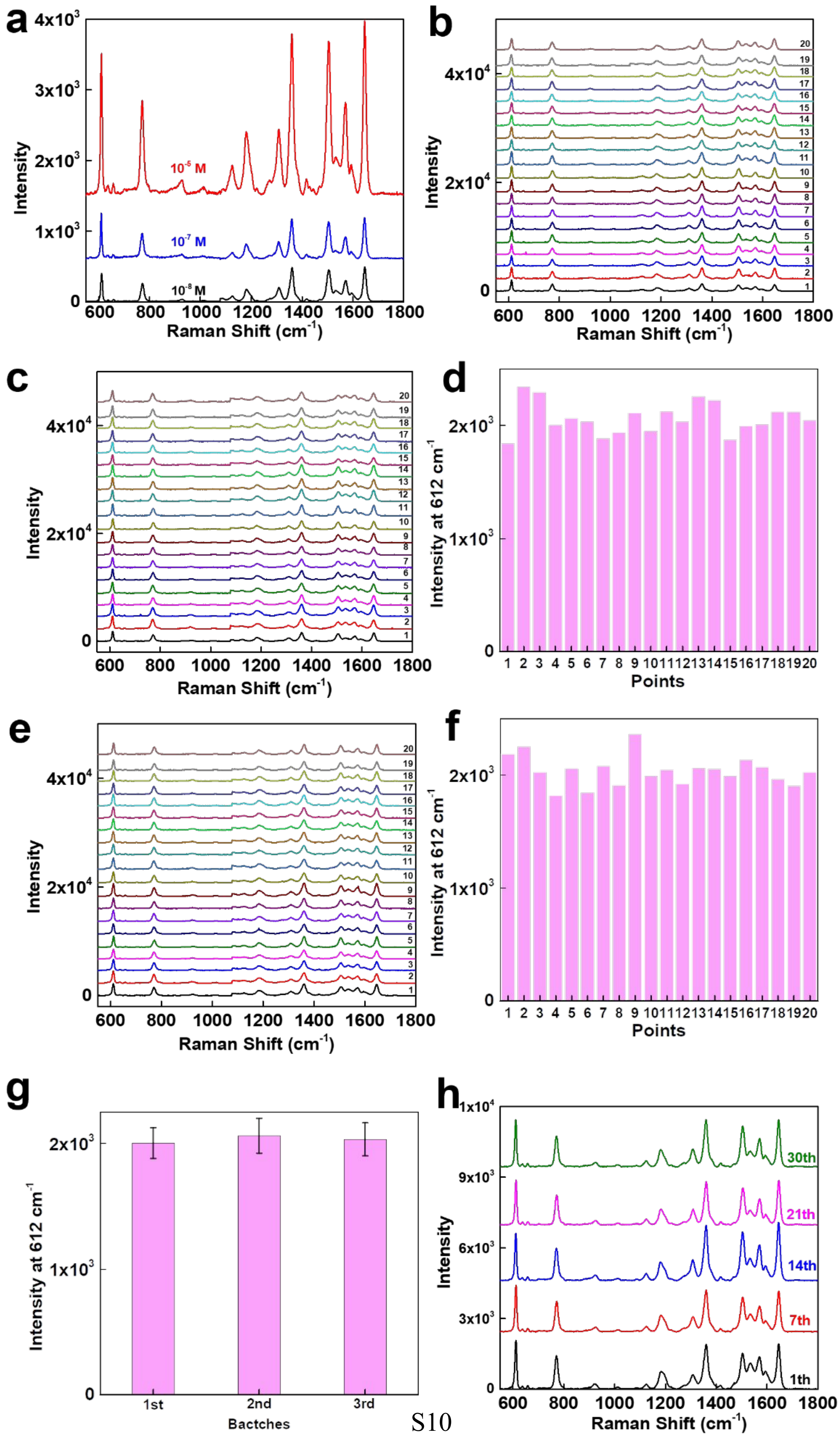
a
b
c
d
e
f
g
h
Intensity
Raman Shift (cm-1)
10-5 M
10-7 M
10-8 M
Intensity at 612 cm-1
Points
Batches
1st
2nd
3rd
1th
7th
14th
21th
30th

Figure S12 (a) SERS spectra of R6G (different concentration from 10^{-8} to 10^{-5} M) with the 100 nm AuNPs. (b) Uniformity test: R6G SERS Raman spectra (612 cm^{-1}) with 100 nm AuNPs at random locations. RSD of 6.1% shows the uniformity of SERS response on 100 nm AuNPs. (c–e) SERS spectra recorded from two independently synthesized AuNP batches prepared on different days under identical R6G conditions. (f) Corresponding intensity distribution at 612 cm^{-1} for the uniformity test, showing low signal fluctuation. (g) Comparison of the average SERS intensities at 612 cm^{-1} for the three batches, demonstrating inter-batch reproducibility. (h) Time-stability study: R6G SERS Raman spectra (612 cm^{-1}) with 100 nm AuNPs at different days.

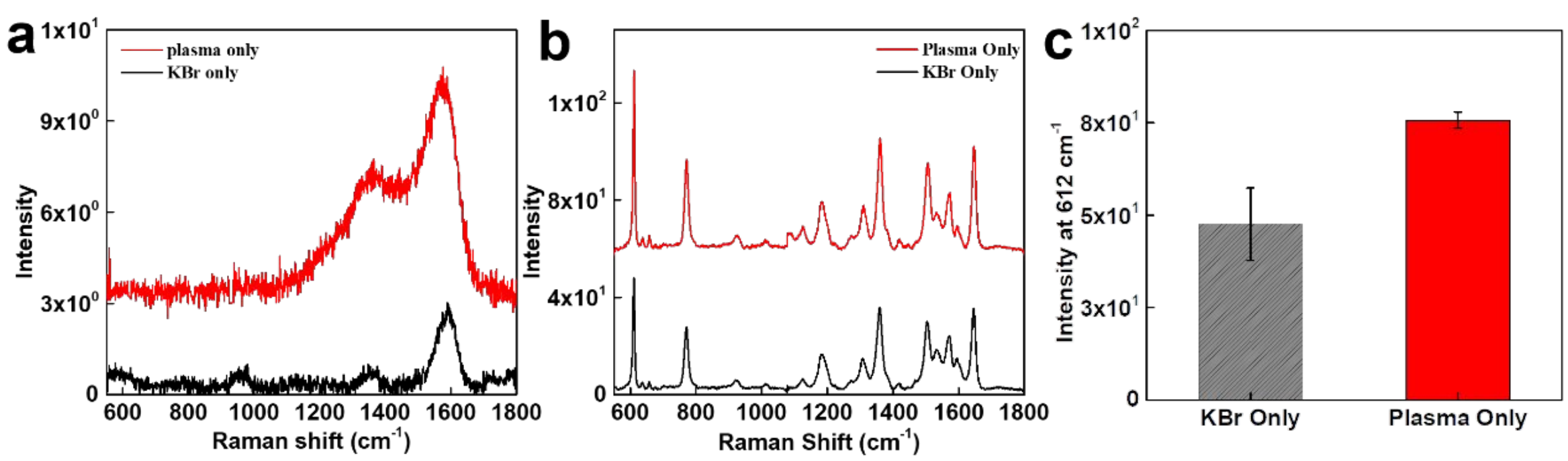

Figure S13 SERS performance of AuNPs treated with plasma only and KBr only. (a) Background Raman signal. (b) Raman spectra showing the characteristic peaks of R6G. (c) Comparison of SERS intensities at 612 cm^{-1}.

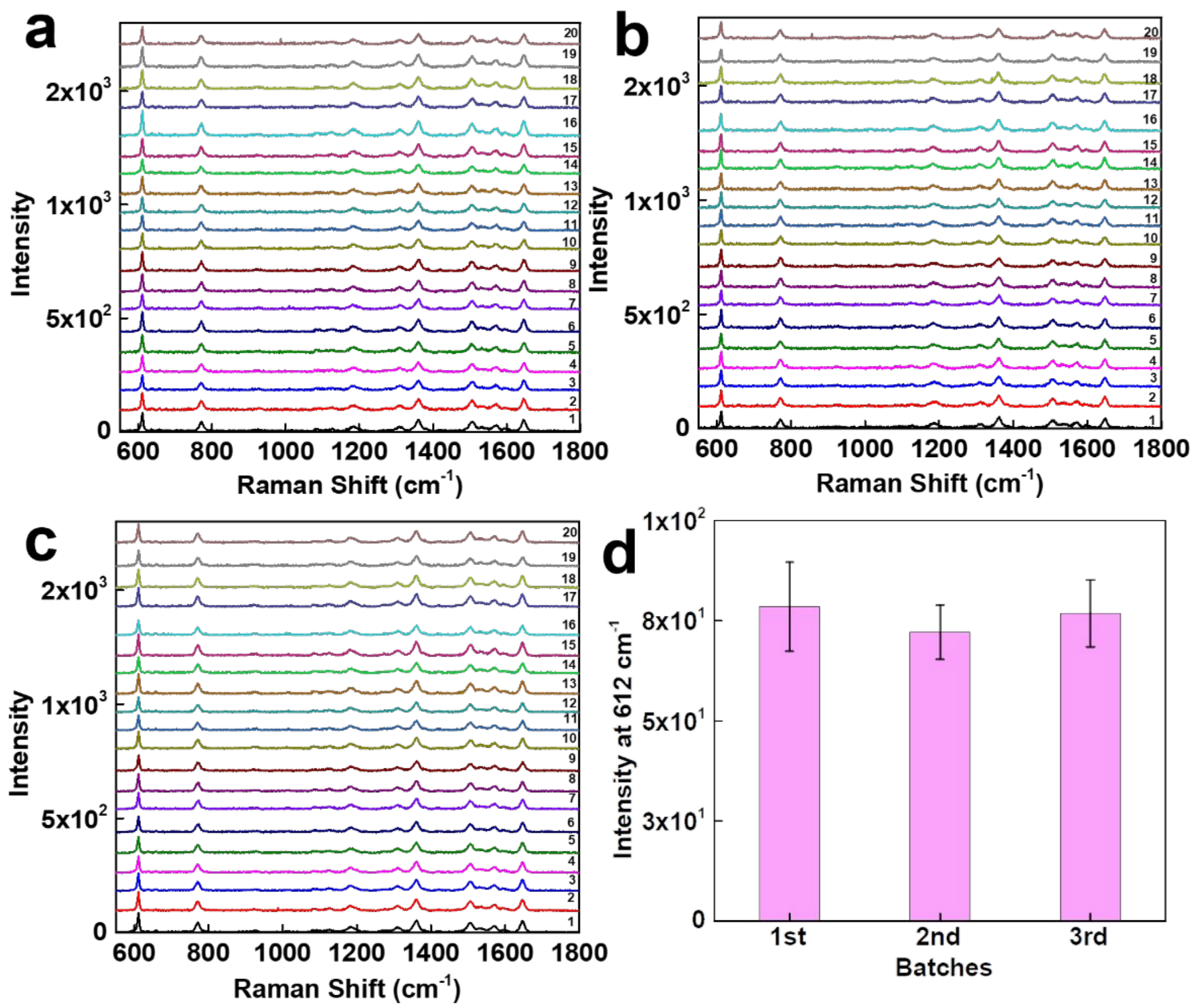

Figure S14 SERS performance of 14 nm AuNPs synthesized via plasma treatment. (a-c) Representative SERS spectra of 10^{-4} M R6G from three independent batches of AuNP substrates (d) Statistical analysis of the SERS intensities at 612 cm^{-1} across the three batches. Error bars represent the standard deviation of the 20 measurements per batch.

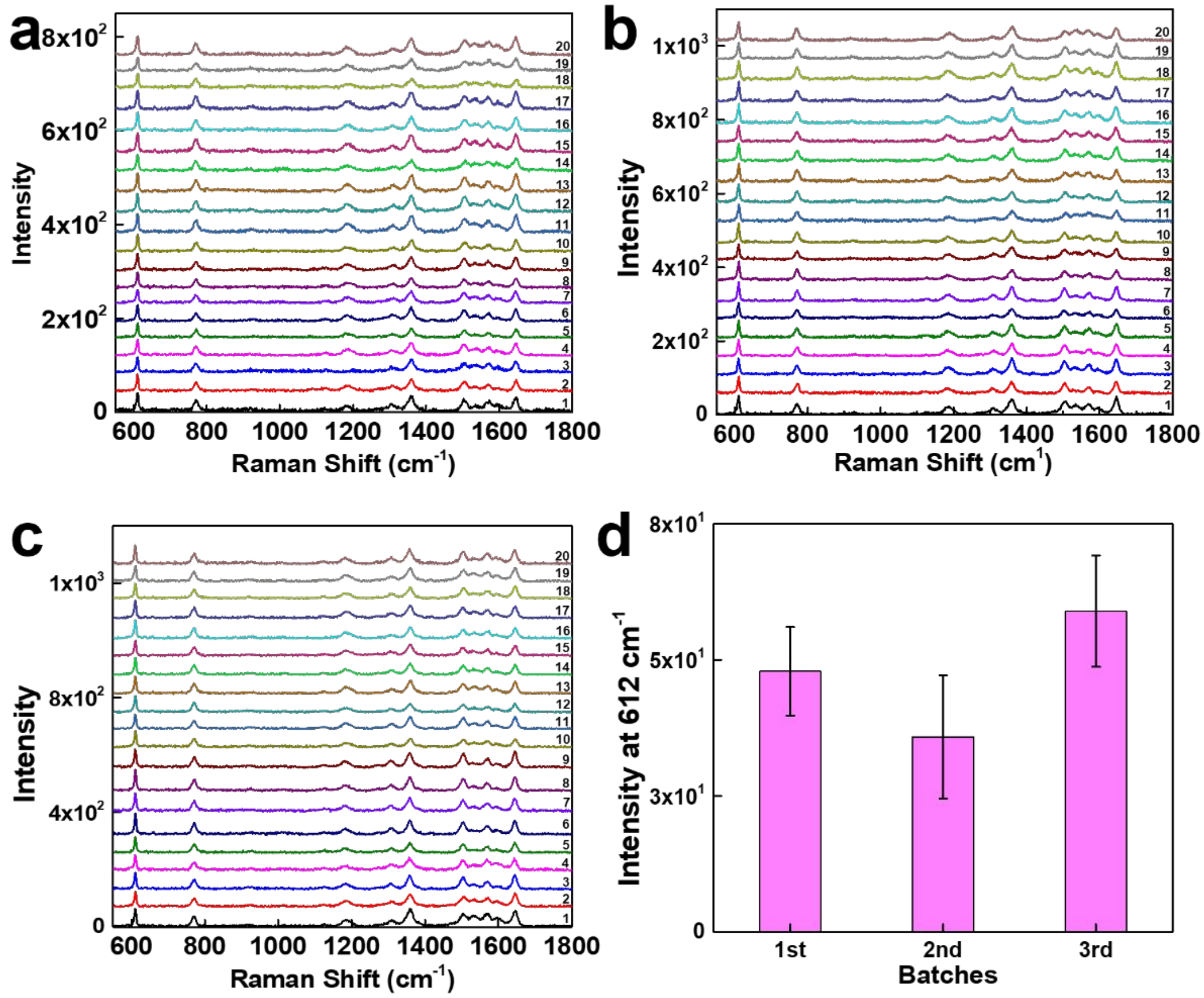

Figure S15 SERS performance of AuNPs synthesized via the traditional citrate reduction method. (a–c) Representative SERS spectra of 10^{-4} M R6G collected from three independent batches, with 20 random measurement spots for each batch. (d) Statistical analysis of the SERS intensity at 612 cm^{-1} across the three batches.

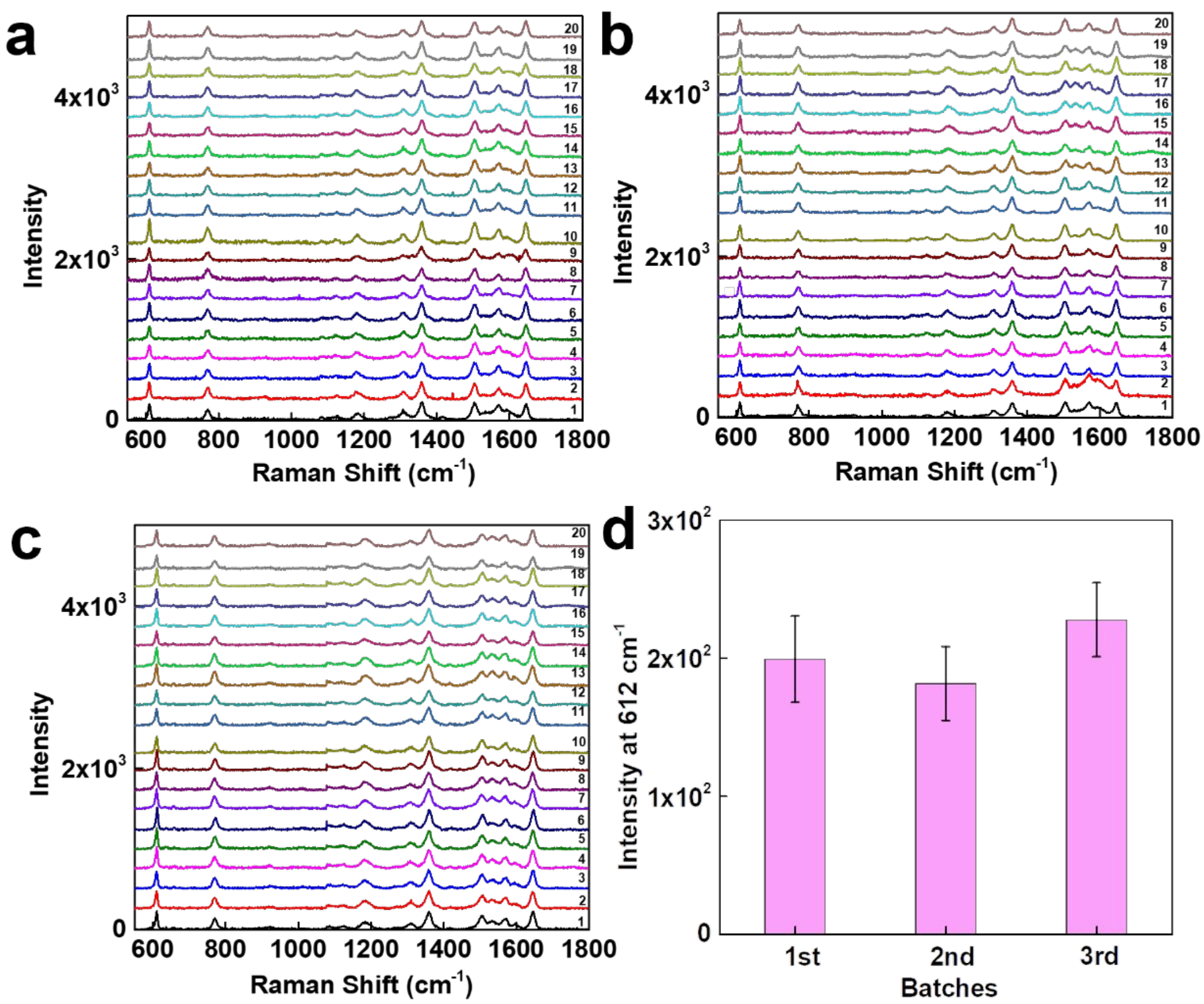

Figure S16 Batch-to-batch SERS reproducibility of KBr-treated 100 nm AuNPs. (a–c) SERS spectra of 10^{-9} -M R6G collected from 20 random spots across three independent batches. (d) Average SERS intensity at 612 cm^{-1} for each batch. Error bars indicate the standard deviation.

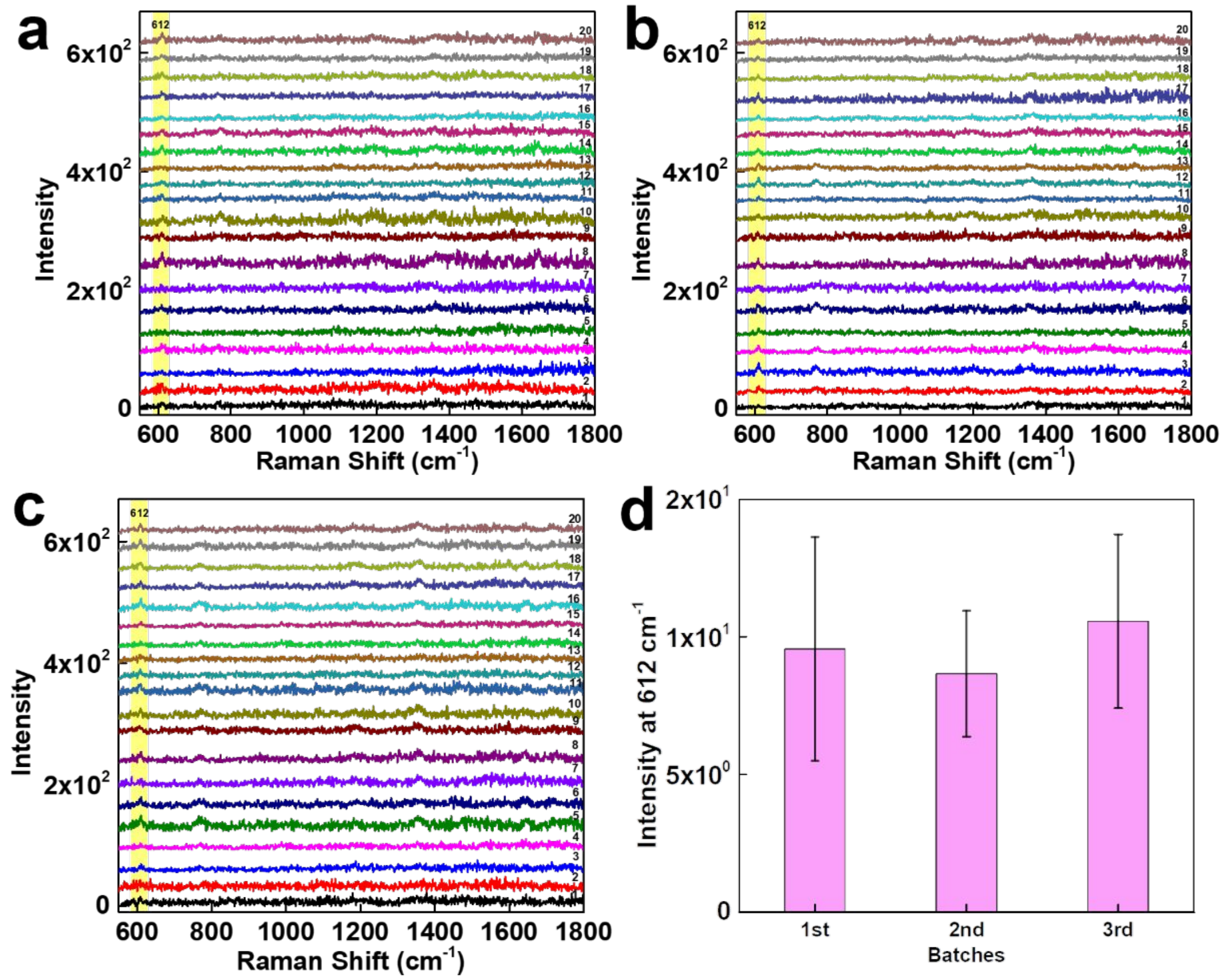

Figure S17 Control measurements of 10^{-4} M R6G on a silicon wafer. (a–c) Raman spectra from 20 random spots in three separate batches. (d) Comparison of the average intensity at 612 cm^{-1} for the three batches (error bars: standard deviation), demonstrating the lack of signal enhancement.

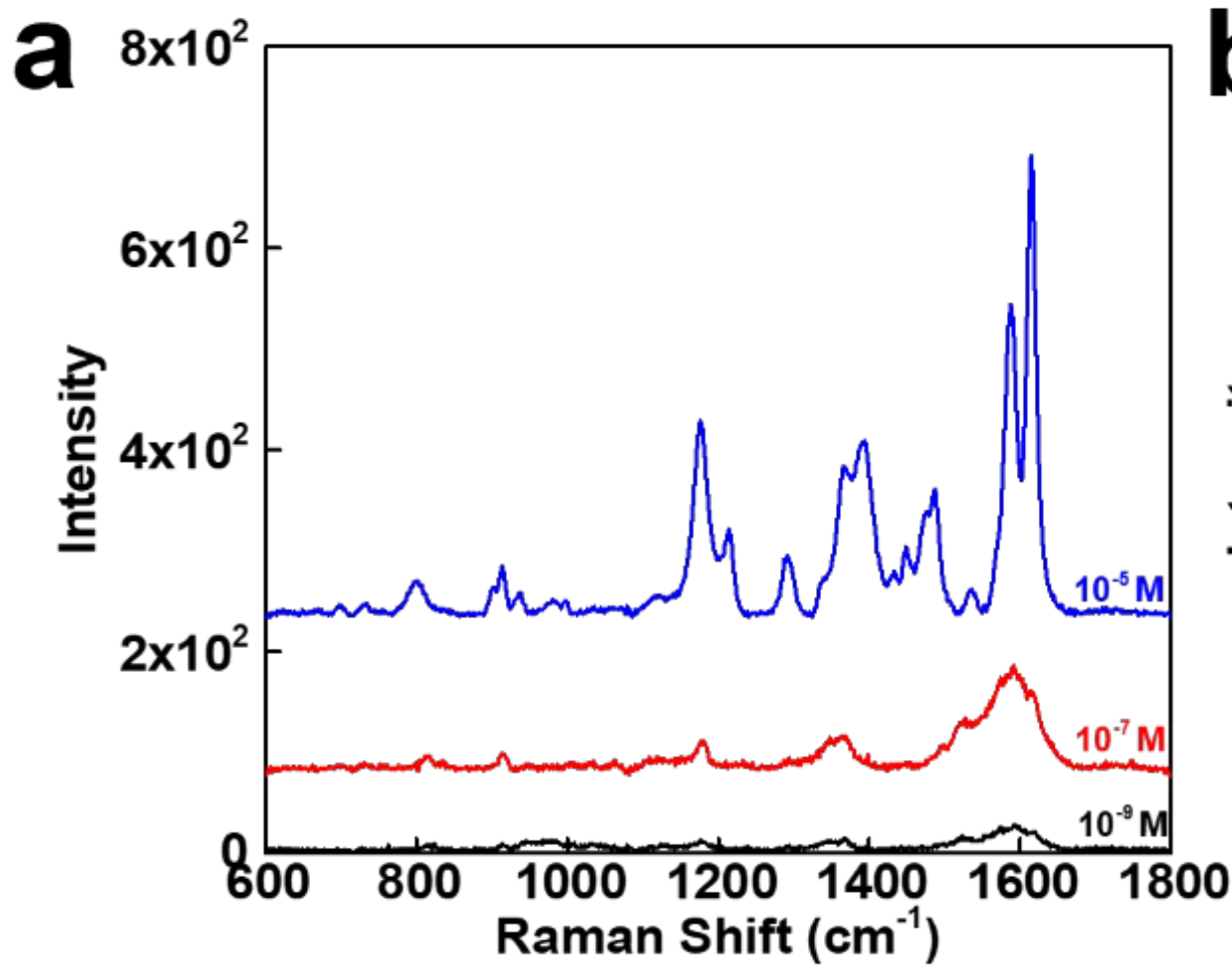

Figure S18 (a) SERS spectra of MG (different concentration from 10^{-9} to 10^{-5} M) with the 100 nm AuNPs. (b) Uniformity test: MG SERS Raman spectra (1615 cm^{-1}) with 100 nm AuNPs at random locations.

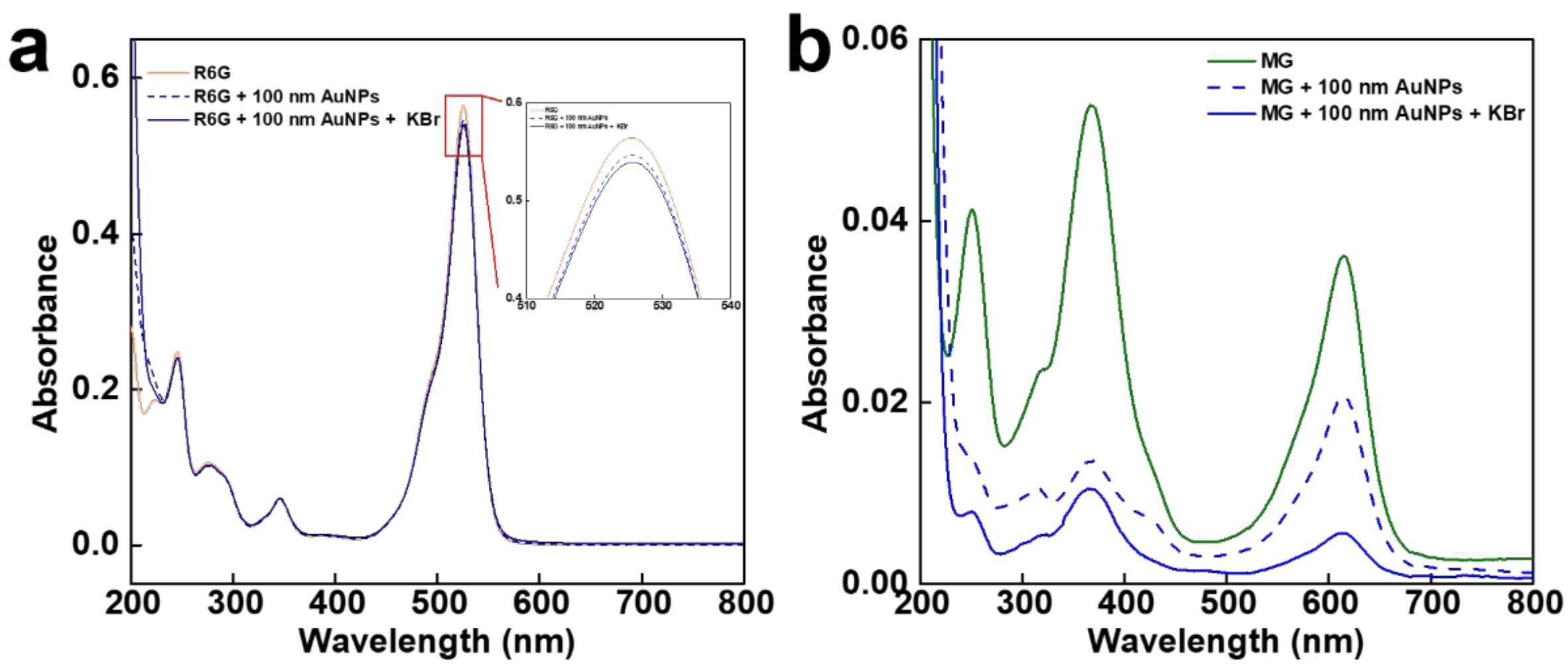

Figure S19 (a)UV-Vis spectra of R6G with 100 nm AuNPs before and after KBr treatment. (b) UV-

Vis spectra of MG with 100 nm AuNPs before and after KBr treatment.

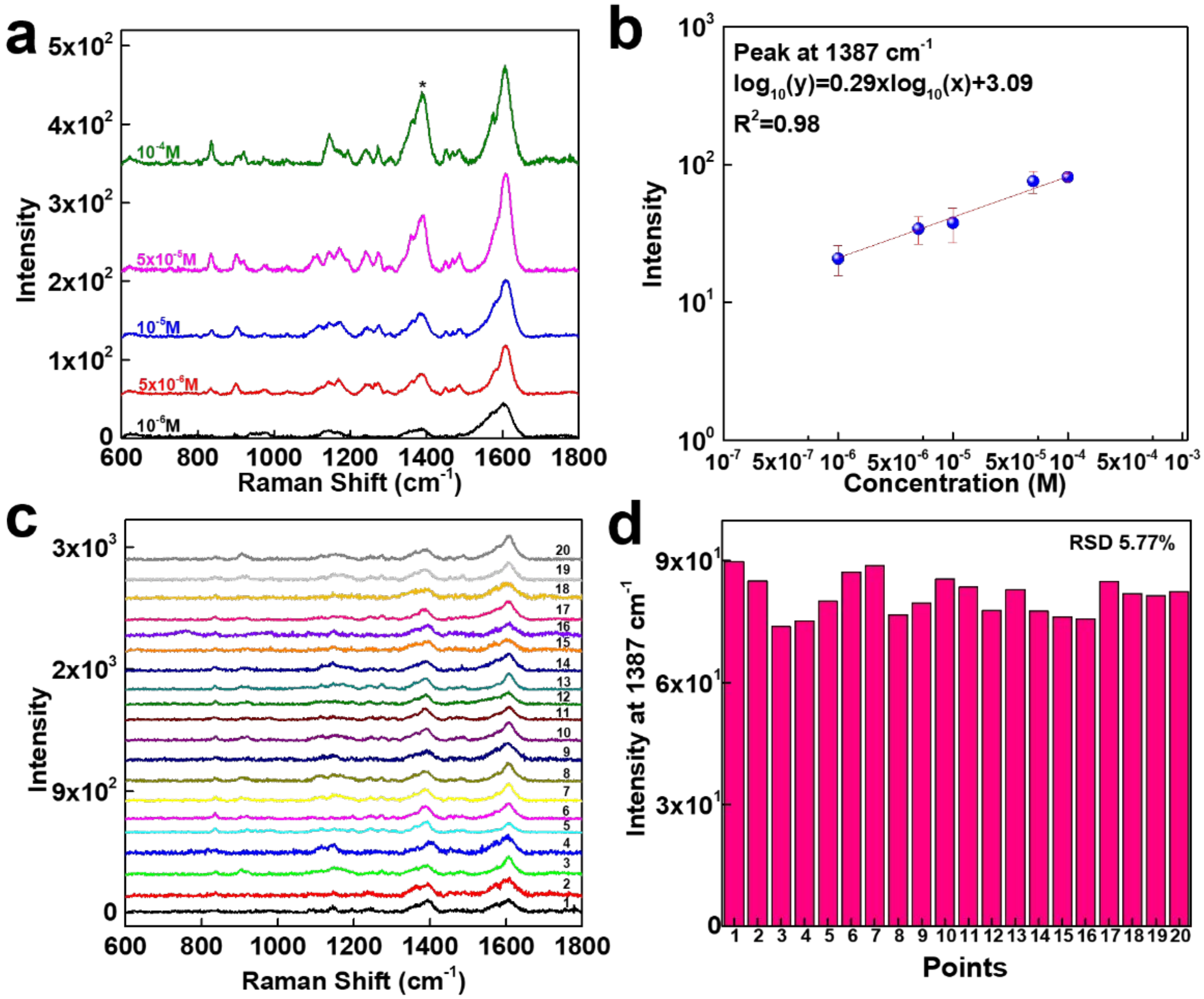

Figure S20 (a) SERS spectra of MR (different concentration from 10^{-6} to 10^{-4} M) with the 100 nm AuNPs. (b) Linear relationship between SERS intensity at 1387 cm^{-1} and MR concentration (from 10^{-6} to 10^{-4} M) using 100 nm AuNPs. (c) and (d) Uniformity test: MR SERS Raman spectra (1387 cm^{-1}) with

100 nm AuNPs at random locations.

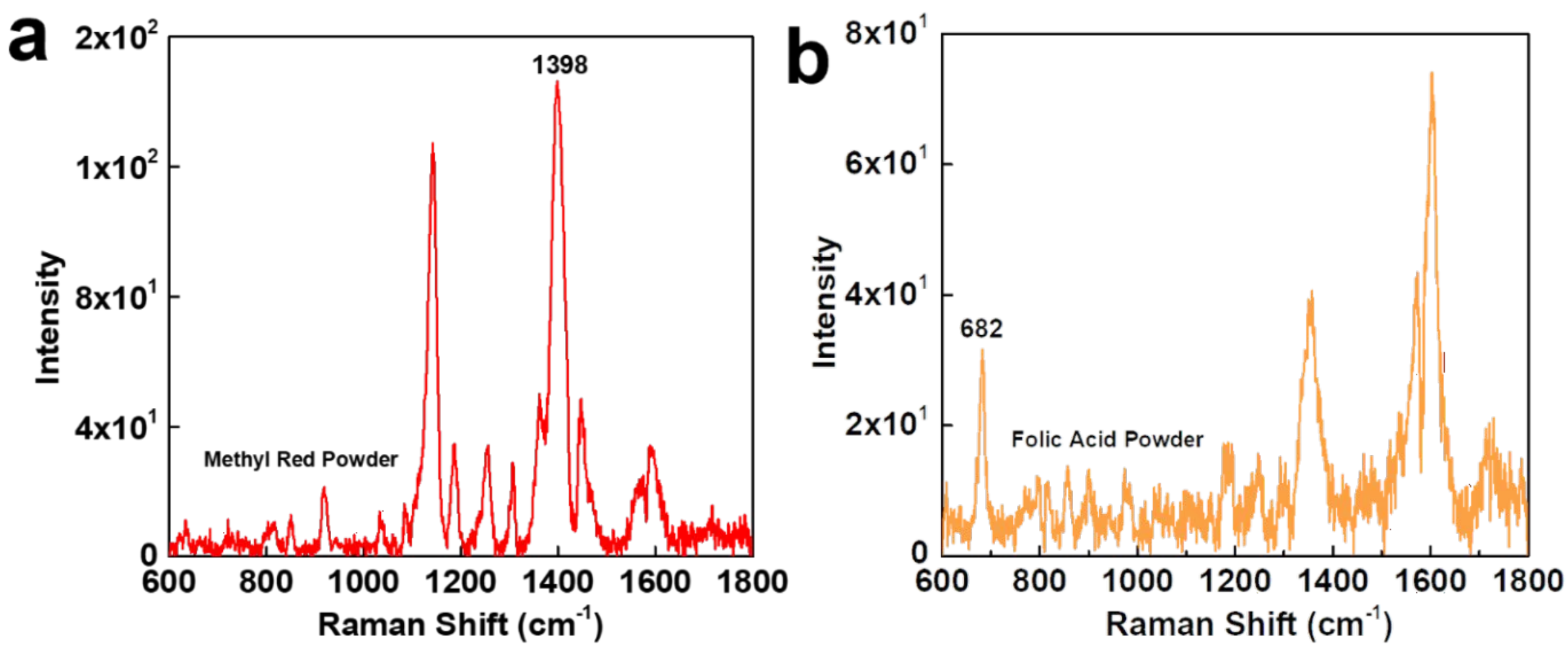

Figure S21 Raman spectra of (a) powder of the MR and (b) powder of the FA powder.

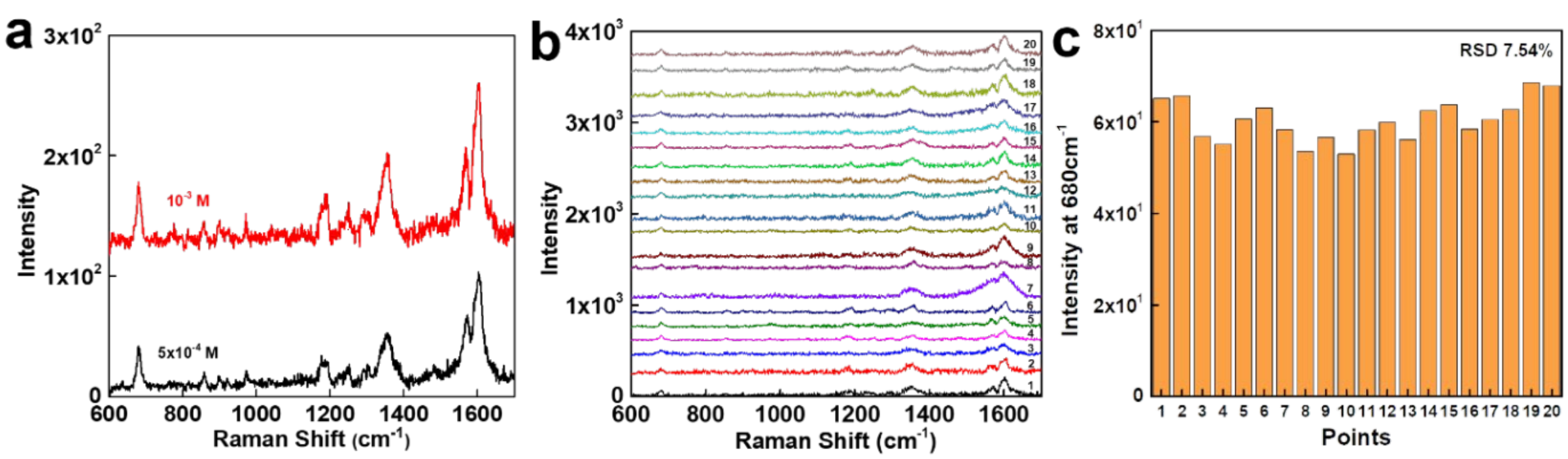

Figure S22 (a) SERS spectra of FA (different concentration from 5×10^{-4} to 10^{-3} M) with the 100 nm AuNPs. (b) and (c) Uniformity test: FA SERS Raman spectra (680 cm^{-1}) with 100 nm AuNPs at random locations.

Table S1 XPS binding energies (eV) and corresponding surface chemical species assignments for 14 nm AuNPs treated with KBr for 0, 1, 2, and 3 hours.

Sample	Surface chemical species (C 1s)		Surface chemical species (O 1s)		Surface chemical species (Au 4f $_{5/2}$)		Surface chemical species (Au 4f $_{7/2}$)		Surface chemical species (Br 3d)	
	B.E.(eV)	Species	B.E.(eV)	Species	B.E.(eV)	Species	B.E.(eV)	Species	B.E.(eV)	Species
**0 hr**	283.2	C=C	529.5	O-Au	86.6	Au^0	82.9	Au^0	-	-
	284.8	C-C/C-H	531.2	O=C						
	286.3	C-O	532.4	C-OH						
	288.3	C=O	535.7	condensed water	87.1	Au^0	83.4	Au^0	-	-
	289.5	O-C-O								
**1 hr**	283.2	C=C	529.5	O-Au	86.6	Au^0	82.9	Au^0	68.5	Br $3d_{5/2}$
	284.8	C-C/C-H	531.2	O=C						
	286.3	C-O	532.4	C-OH	87.1	Au^0	83.2	Au^0	69.5	Br $3d_{3/2}$
	288.3	C=O	535.7	condensed water						
**2 hr**	283.2	C=C	529.5	O-Au	86.5	Au^0	82.9	Au^0	68.5	Br $3d_{5/2}$
	284.8	C-C/C-H	531.5	O=C						
	286.3	C-O	532.4	C-OH	87.1	Au^0	83.4	Au^0	69. 5	Br $3d_{3/2}$
	288.3	C=O	535.7	condensed water						
**3 hr**	283.2	C=C	529.5	O-Au	87.1	Au^0	83.4	Au^0	68.5	Br $3d_{5/2}$
	284.8	C-C/C-H	531.3	O=C						
	286.3	C-O	532.4	C-OH	88.0	Au^0	84.3	Au^0	69.5	Br $3d_{3/2}$
	288.3	C=O	535.7	condensed water						

Table S2 Atomic percentage summary of the synthesized 14 nm AuNPs mixed with KBr at different times.

Atomic Percentage obtained from XPS measurement (at%)				
**Sample**	**C**	**O**	**Au**	**Br**
**0 hr**	56.08	43.61	0.31	-
**1 hr**	64.04	34.23	0.51	1.22

**2 hr**	56.66	41.49	0.49	1.36
**3 hr**	69.31	28.86	0.88	0.95

Table S3 Bonding percentage summary of the synthesized 14 nm AuNPs mixed with KBr at different times.

**Bonding percentage obtained from C1s fitting (%)**					
**Sample**	C=C	C-C/C-H	C-O	C=O	condensed water
**0 hr**	0.81	34.55	7.26	12.77	0.69
**1 hr**	1.47	46.83	7.01	8.73	–
**2 hr**	1.27	39.68	6.36	9.35	–
**3 hr**	1.28	52.89	6.95	8.19	–

**Bonding percentage obtained from Au4f fitting (%)**				
**Sample**	Au $4f_{7/2}$	Au $4f_{5/2}$	Au $4f_{7/2}$	Au $4f_{5/2}$
**0 hr**	0.10	0.08	0.07	0.06
**1 hr**	0.10	0.17	0.12	0.12
**2 hr**	0.14	0.13	0.10	0.12
**3 hr**	0.32	0.18	0.27	0.11

**Bonding percentage obtained from O1s fitting (%)**				
**Sample**	O-Au	O=C	C-OH	condensed water
**0 hr**	0.45	21.32	12.78	9.06

**1 hr**	0.87	15.89	11.57	5.90
**2 hr**	0.95	20.33	12.13	8.08
**3 hr**	0.77	10.40	12.84	4.85

Bonding percentage obtained from Br3d fitting (%)

**Sample**	Br $3d_{5/2}$	Br $3d_{3/2}$
**1 hr**	0.97	0.25
**2 hr**	0.89	0.47
**3 hr**	0.51	0.44

Table S4 Vibration mode description of characteristic peaks in Raman spectra of R6G.

**Raman vibration mode**	**Raman shift (cm^{-1})**
C−S stretching	612
C-C bond stretching	722
in-plane C–H vibrations	1182
	1360
aromatic C–C bonds	1506
	1647

Table S5 Average SERS intensity (I_{SERS}) of 10^{-9} M R6G on KBr-treated 100 nm AuNPs and unenhanced normal Raman intensity (I_{Raman}) of 10^{-4} M R6G on a bare silicon wafer, collected from three independent batches for the Analytical Enhancement Factor (AEF) calculation.

	1st	2nd	3rd	Average
I_{SERS}	199.11±31.20	181.47±26.75	227.54±26.76	202.71±28.23
I_{Raman}	9.55±4.07	8.65±2.28	10.56±3.16	9.58±3.17

Table S6 SERS intensities at 612 cm⁻¹ for plasma-treated and KBr-only AuNPs, reported as mean ± standard deviation from 20 measurements per batch across three independent batches.

	1st	2nd	3rd	Average
$I_{Surf.A}$ (plasma only)	78.41±11.13	72.05±6.77	76.72±8.37	75.72±2.20
$I_{Surf.A}$ (KBr only)	47.9±8.12	35.8±11.30	58.95±10.24	47.55±9.80
$I_{Surf.B}$	9.55±4.07	8.65±2.28	10.56±3.16	9.58±3.17

Table S7 Vibration mode description of characteristic peaks in Raman spectra of MG.

**Raman vibration mode**	**Raman shift (cm^{-1})**
C–H out-of-plane bending (wagging)	911
In-plane C–H bending	1173
Aromatic C–C stretching;	1364
Symmetric phenyl–N stretching	1384
Aromatic C=C stretching	1586

Aromatic C=C stretching	1615

Table S8 Performance of the SERS biosensor for the MG detection.

Materials	LoD (M)	Reference
CNT-g-PAA/AuNRs	10^{-10}	2
AuNPs@MIL-100 (Fe)	3.04×10^{-10}	3
$AuNPs/TiO_2/Ti_3C_2$ Heterostructures	2.71×10^{-10}	4
Au NP-MLF	10^{-8}	5
80 nm AuNPs on silicon Reusable Substrates	10^{-11}	6
Monolithic gold saturated on nanoporous gold@mirror template	1.7×10^{-9}	7
gold–silicon heterogeneous nano pineapple structures	7.8×10^{-11}	8
Au NBPs@ Ag	1.0×10^{-10}	9
Au@Ag Nanorods	1.58×10^{-9}	10
porous carbon microspheres to assemble AuNP	1.0×10^{-10}	11

Table S9 Vibration mode description of characteristic peaks in Raman spectra of MR.

Raman vibration mode	Raman shift (cm^{-1})
C-Cethyl stretching, N-Ring bending	835
C-N-C stretching	920
C-N= stretching	1145
C-Ring stretching	1365
N=N stretching	1387
C, N-Rings stretching	1452
C-Rings stretching	1578
C-ring, C-C stretching	1606

Table S10 Vibration mode description of characteristic peaks in Raman spectra of FA.

Raman vibration mode	Raman shift (cm^{-1})
out-of-plane (C=C) (PABA) stretching, out-of-plane (C=N) (PT) stretching	680
(CH_2) (PABA) rocking	971
(O-H) (PT) bending, (CH2, O-H) (glutamic acid) bending	1186
(C-H, O-H) (glutamic acid) rocking, (CH_2) (glutamic acid) wagging	1250
(C-C, C-N, C-O) (PT) stretching	1355
(C-NH_2, C-C, C=N) (PT) stretching, (NH_2) (PT) bending	1568
(NH2) (PT) scissoring, (C=N) (PT) stretching, (C=C)(PABA) stretching, (CH) (PABA) rocking	1604

References

(1) Alvarez-Puebla, R.; Cui, B.; Bravo-Vasquez, J.-P.; Veres, T.; Fenniri, H. Nanoimprinted SERS-Active Substrates with Tunable Surface Plasmon Resonances. *J. Phys. Chem. C* **2007**, *111* (18), 6720-6723.

(2) Geng, L.; Han, X.; You, J.; Tian, Y.; Yang, J.; Xu, H.; Yang, W.; Xu, H. Oriented Gold Nanorods on Polyacrylic Acid Grafted Carbon Nanotube for Efficient SERS Detection of Cationic Dyes. *Spectrochim. Acta, Part A* **2025**, 125871.

(3) Han, H.; Huang, Y.; Miao, J.; Lai, K. Rapid Determination of Malachite Green in Fish by Surface-Enhanced Raman Spectroscopy Combined with Mil-100 (Fe). *Microchim. Acta* **2025**, *192* (3), 185.

(4) Fang, T.; Wei, Q.; Wu, E.; Pu, H. Elevating Electron Transfer of Recyclable SERS Sensor Using AuNPs/TiO2/Ti3C2 Heterostructures for Detection of Malachite Green in Sunfish. *Spectrochim. Acta, Part A* **2025**, *325*, 125047.

(5) Zhang, B.; Li, L.; Zhang, R.; Guo, Y.; Fang, J. A Molecular Detection Strategy Via Confined-Enhanced Raman Spectroscopy on a Self-Assembled Au Nanoparticle Film. *ACS Appl. Nano Mater.* **2025**.

(6) Du, W.; Zhao, B.; Lun, Y.; Hu, Y.; Geng, Y.; Zhou, Y.; Zhao, X.; Wang, X. Fabrication of Silicon Surface Microstructures Via Vortex Femtosecond Laser Irradiation for Reusable Substrates in SERS Applications. *Small* **2025**, 2410480.

(7) Ahmad, W.; Wang, J.; Wu, X.; Zareef, M.; Adade, S. Y.-S. S.; Xu, Y.; Chen, Q. Monolithic Gold Saturated on Nanoporous Gold@ Mirror Template for Highly Reliable and Fast Surface-Enhanced Raman Scattering Detection. *Biosens. Bioelectron.* **2025**, *268*, 116929.

(8) Liu, Y.-n.; Li, J.-j.; Weng, G.-j.; Zhu, J.; Zhao, J.-w. Reliable Detection of Malachite Green by Self-Assembled SERS Substrates Based on Gold–Silicon Heterogeneous Nano Pineapple Structures. *Food Chem.* **2024**, *451*, 139454.

(9) Niu, F.; Jiang, P.; Wang, L.; Gao, Y.; Zhu, H.; Gao, N.; Cai, Z.; He, H.; He, Y.; Chang, G.

Controllable Growth of Au NBPs@ Ag Nanorods Modified PDMS as SERS Substrate for Rapid Detection of Malachite Green in Aquatic Products. *Microchem. J.* **2024**, *201*, 110569.

(10) Zhou, X.; Chen, S.; Pan, Y.; Wang, Y.; Xu, N.; Xue, Y.; Wei, X.; Lu, Y. High-Performance Au@ Ag Nanorods Substrate for SERS Detection of Malachite Green in Aquatic Products. *Biosensors* **2023**, *13* (8), 766.

(11) Hinds, D. T.; Belhout, S. A.; Colavita, P. E.; Ward, A. D.; Quinn, S. J. Microsphere-Supported Gold Nanoparticles for SERS Detection of Malachite Green. *Mater. Adv.* **2023**, *4* (6), 1481-1489.

.
